# Supplementary material for: Van der Waals polarity-engineered 3D integration of 2D complementary logic
Source: Nature. 2024 May 29;630(8016):346–52. doi: 10.1038/s41586-024-07438-5 (PMC11168927; doi:10.1038/s41586-024-07438-5)
Supplement: Supplementary file 1 — Supplementary Notes 1–4, Figs 1–30, Tables 1–3 and References. [file 41586_2024_7438_MOESM1_ESM.pdf]

nature portfolio

https://doi.org/10.1038/s41586-024-07438-5

Supplementary information

Van der Waals polarity-engineered 3D integration of 2D complementary logic

In the format provided by the authors and unedited

Supplementary Information for

Van der Waals polarity-engineered 3D integration of 2D complementary logics

Yimeng Guo, Jiangxu Li, Xuepeng Zhan, Chunwen Wang, Min Li, *et al.*

Outlines

1. Supplementary Note 1. Band structure of MoS_2, CrOCl, and MoS_2/CrOCl heterostructures

2. Atomic force microscopy (AFM) characterization of a typical MoS_2/CrOCl p-type field effect transistor (p-FET)

3. Statistics of transfer characteristics of MoS_2/CrOCl FETs

4. Output characteristics of a typical MoS_2/CrOCl p-FET

5. Kelvin probe force microscopy (KPFM) characterizations of MoS_2/CrOCl heterostructures

6. Raman spectra of a typical MoS_2/CrOCl sample

7. Electrical performance of $MoSe_2$/CrOCl and WSe_2/CrOCl FET

8. Electrical performance of MoS_2/CrOCl based planar inverter

9. Noise margin of the MoS_2/CrOCl based vertical complementary FET (CFET) logic inverter

10. Dynamic inverting performance of the MoS_2-based vertical CFET inverter

11. Estimation of field effect hole mobilities in typical devices

12. Performances of NAND logic based on MoS_2/CrOCl VIP-FETs

13. Supplementary Note 2. Discussions of universality of the interfacial coupling induced p-doping effect and the improvement of their electrical performances

14. Supplementary Note 3. Possibility of large-scale production of CrOCl thin films

15. Supplementary Note 4. Technology Computer-Aided Design (TCAD) Simulations of parasitic capacitances of the VIP-FETs devices

1. Supplementary Note 1. Band structure of MoS_2/CrOCl heterostructures

In this Supplementary Note, we give details of the density functional theory (DFT) calculations as described in the main text in the section "Modeling of vdW polarity-engineered MoS_2". Notice that Fig. 1d in the main text is a focused interface calculated from a 3-layered MoS_2 + 3-layered CrOCl heterostructure, and the holes located on the MoS_2 side of the interface are compensated by electrons deep in MoS_2 so that the entire MoS_2 multi-layer remains charge neutral, and almost all the doped electron carriers are transferred to CrOCl. Here, to test the interfacial charge-transfer effects, additional electron charges (~ 10^{11} cm^{-2}) are injected into the system under zero electric field. MoS_2 still remains charge neutral since no electric field has been applied, and the chemical potential remains in the gap of MoS_2.

Calculation methods including the single particle and interaction pictures can be found in the Methods part in the main text. Here, we discuss some additional detailed results. In Supplementary Figure 1b and d, we show the band structures of MoS_2 and CrOCl, respectively. Their lattice structures are given in Supplementary Figure 1a and c. We consider two competing magnetic states of CrOCl: (I) the interlayer antiferromagnetic (AFM) and intralayer ferromagnetic (FM) state as shown in Supplementary Figure 1e, and interlayer and intralayer AFM state as shown in Supplementary Figure 1f. It turns out that the magnetic ground state of CrOCl in CrOCl-MoS_2 heterostructure is the type (II) state. We adopt type (II) magnetic order in all of our calculations.

In Supplementary Figure 2a-b we show the side view and top view of the lattice structure of MoS_2-CrOCl heterostructure. Supplementary Figure 3a shows the electronic band structures of MoS_2-CrOCl heterostructure under zero electric field. The contributions from MoS_2 and CrOCl orbitals are marked by blue and red colors, respectively. We see that the MoS_2 bands have little hybridization with those from CrOCl. The conduction band minimum (CBM) of the heterostructure is contributed by CrOCl valence bands and the valence band maximum (VBM) is contributed by MoS_2 conduction bands.

We have calculated the band structures of the MoS_2/CrOCl heterostructure, from which the band alignment can be treated more accurately. We find that, in the absence of vertical electric fields, the heterostructure supercell calculation indicates that the CBM of CrOCl is 0.466 eV lower than the CBM of MoS_2, and is 0.488 eV above the valence band maximum (VBM) of MoS_2, as schematically shown in Supplementary Figure 4b. This is qualitatively consistent with those obtained from work-function calculations. Moreover, we find that the effective mass around CBM of CrOCl is m^*= 2.88 m_e (m_e is the bare electron mass). If a small amount of electron carriers (~10^{12} cm^{-2} as deduced from the applied gate voltage) are transferred to the

surface of CrOCl, the corresponding Wigner-Seitz radius is as large as 122 (assuming a dielectric constant $\varepsilon = 5$), far above the threshold value to form a Wigner crystal. This implies that the small amount of electron carriers transferred to CrOCl would not conduct, rather they tend to spontaneously crystallize driven by long-range *e-e* interactions and form an insulating electronic crystal.[1] This explains why the system remains insulating even under nominal electron doping.

We have also performed first principles DFT calculations for 10-layer slab of MoS_2 and 5-layer slab of CrOCl with electric fields (***E***) ranging from - 0.2 V/nm to 0 V/nm (negative electric fields correspond to negative bottom gate voltages in our experimental configurations). The evolution of the CBM and VBM of CrOCl and MoS_2 with respect to vacuum level are given in Supplementary Table 1. When ***E*** is negative, the CBM of CrOCl slightly decreases with respect to that of zero electric field, corresponding to the case of negative gate voltage in our experimental set up. In the meanwhile, the VBM of MoS_2 increases rapidly under negative electric field. For example, when ***E*** = - 0.15 V/nm, the CBM of CrOCl has been lowered by 0.16 eV compared to that with zero electric field, while the VBM of MoS_2 is dramatically increased in energy by 0.25 eV (compared to the zero-field case), which is only 0.074 eV below CrOCl CBM, as shown in Supplementary Figure 3b (also see schematic illustration in Supplementary Figure 4c). As a result, if the system is initially electron doped, the electron carriers would be transferred to the CBM of CrOCl, and the chemical potential is only about 0.074 eV above the VBM of MoS_2. Furthermore, as discussed above, the electron carriers transferred to the surface of CrOCl occupying its CBM are expected to be frozen to form an electronic crystal due to the large effective mass and small transferred carrier density, which would further pull down the chemical potential, thus MoS_2 can be easily hole doped upon further application of negative gate voltage. All the band edges discussed here are those close to the MoS_2/CrOCl interface.

The above calculations with finite electric fields are based on slab models, which can capture the qualitative features of band alignment under vertical electric fields. A more comprehensive and accurate study requires direct first principles DFT calculations based on CrOCl/MoS_2 heterostructures under finite electric fields. More detailed results about this mechanism can be found in our forthcoming theory paper [2].

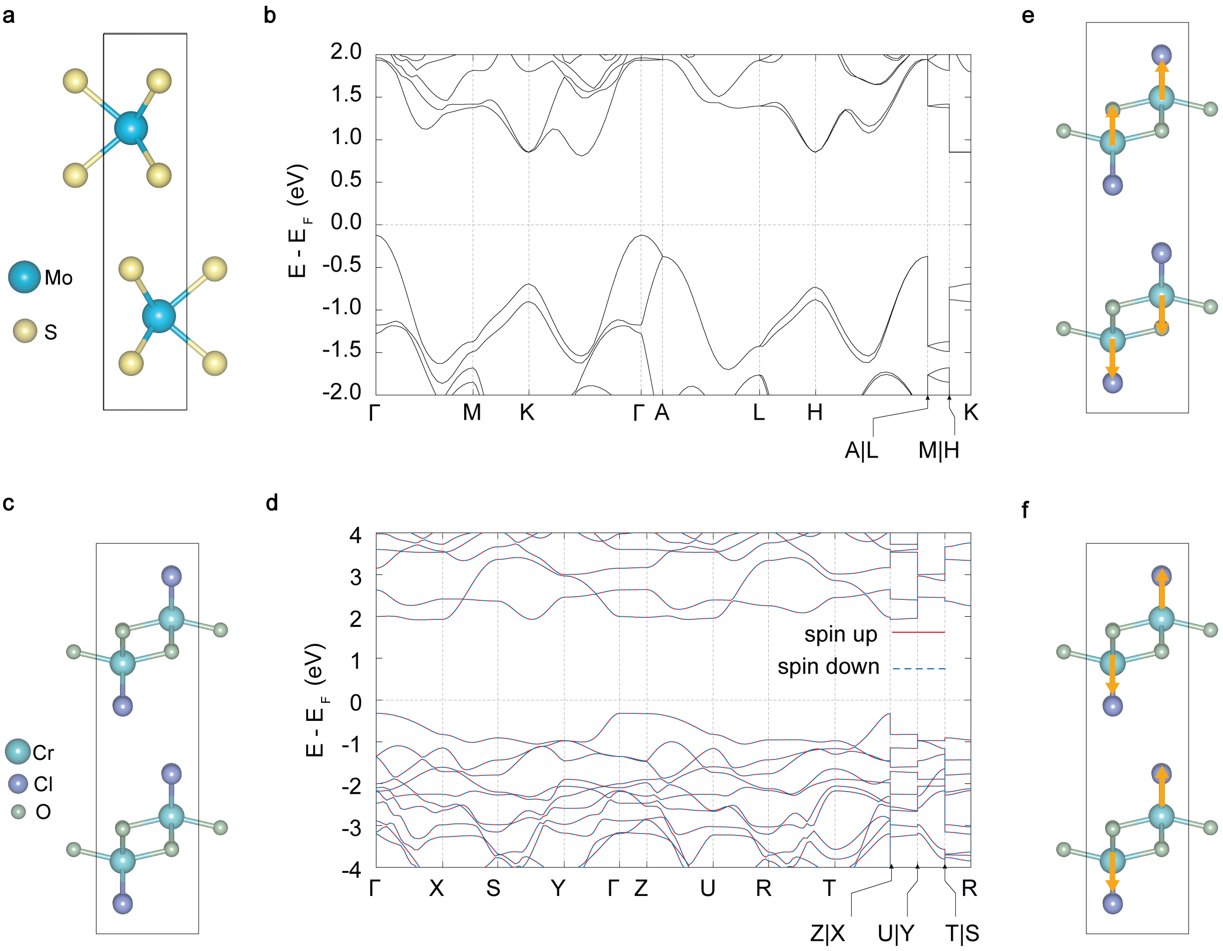

Supplementary Figure 1. Band structures and magnetic configurations of MoS_2 and CrOCl. Crystal structures of (a) MoS_2 and (c) CrOCl. The energy bands of (b) bulk MoS_2 and (d) bulk CrOCl. In order to take into account of the subtle *e-e* interactions, spin configurations of the CrOCl crystal have to be calculated. We here considered two magnetic configurations: (I) an interlayer antiferromagnetic and intralayer ferromagnetic state as shown in (e); and (II) an intralayer and interlayer antiferromagnetic state as shown in (f).

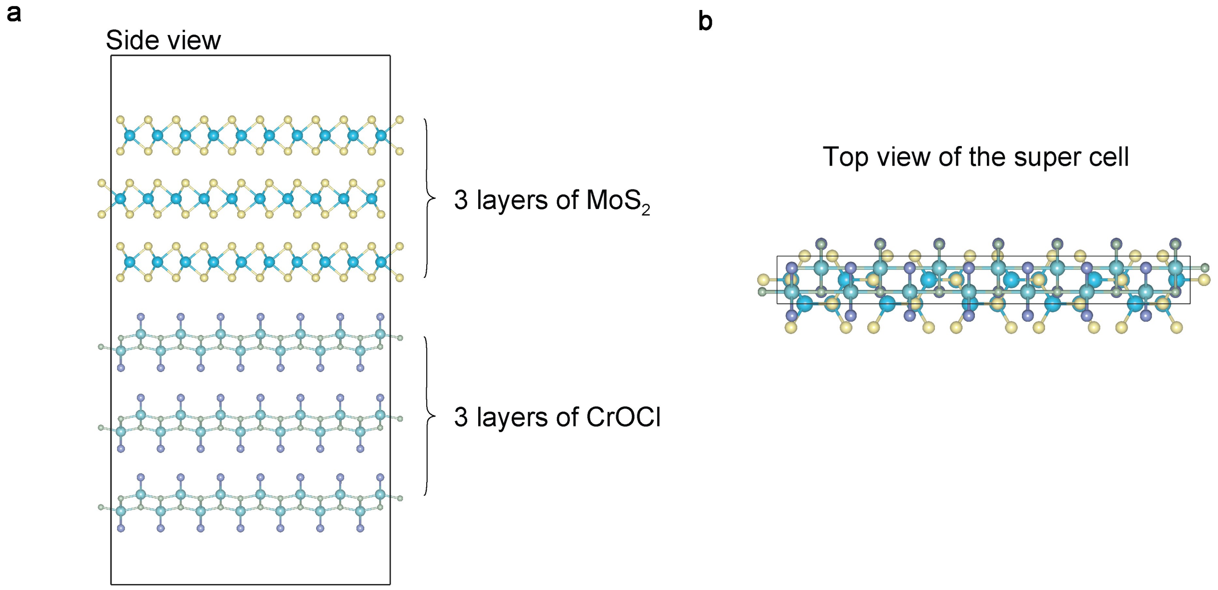

Supplementary Figure 2. Unit cell of the MoS_2-CrOCl heterostructure. (a) and (b) indicate the side and top views of the heterostructure of three layers of MoS_2 on top of three layers of CrOCl, as described in detail in the Methods section in the main text.

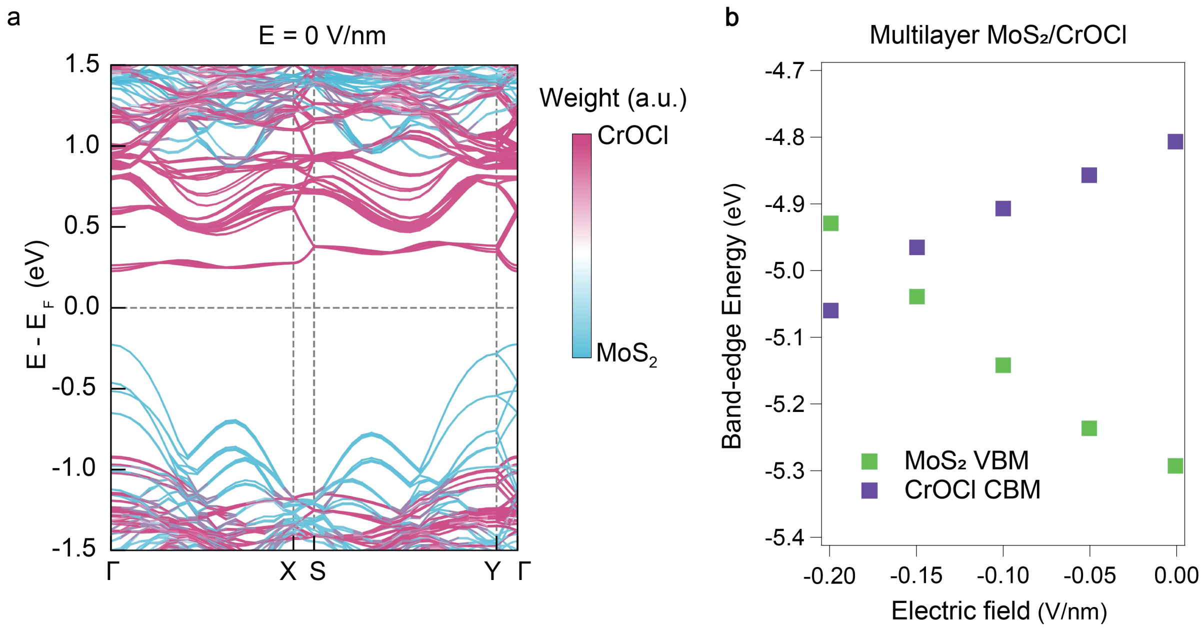

Supplementary Figure 3. Calculated band structure of MoS_2-CrOCl heterostructures. (a) show the electronic band structures of MoS_2-CrOCl heterostructure (shown in Supplementary Figure 2) under zero electric field. The contributions from MoS_2 and CrOCl are marked by blue and red colours, respectively. (b) Calculated band edge of CBM for CrOCl (modelled as 5-layer slab) and VBM for MoS_2 (modelled as 10-layer slab), as a function of electrical fields. Here, negative electric fields correspond to negative bottom gate voltages in our experimental configurations.

Supplementary Table 1. Evolution of CBM and VBM of 10-layer MoS_2 slab and 5-layer CrOCl slab under different electric field ***E*** (in units of V/nm). Vacuum energy is set to zero. The band-edge energies are in units of eV.

E (V/nm)	-0.20	-0.15	-0.10	-0.05	0.00
MoS_2 CBM	-4.8931	-4.7529	-4.6076	-4.4623	-4.3398
CrOCl CBM	-5.0590	-4.9641	-4.9056	-4.8555	-4.8050
MoS_2 VBM	-4.9280	-5.0382	-5.1414	-5.2361	-5.2927
CrOCl VBM	-6.5951	-6.6664	-6.7553	-6.8539	-6.9255

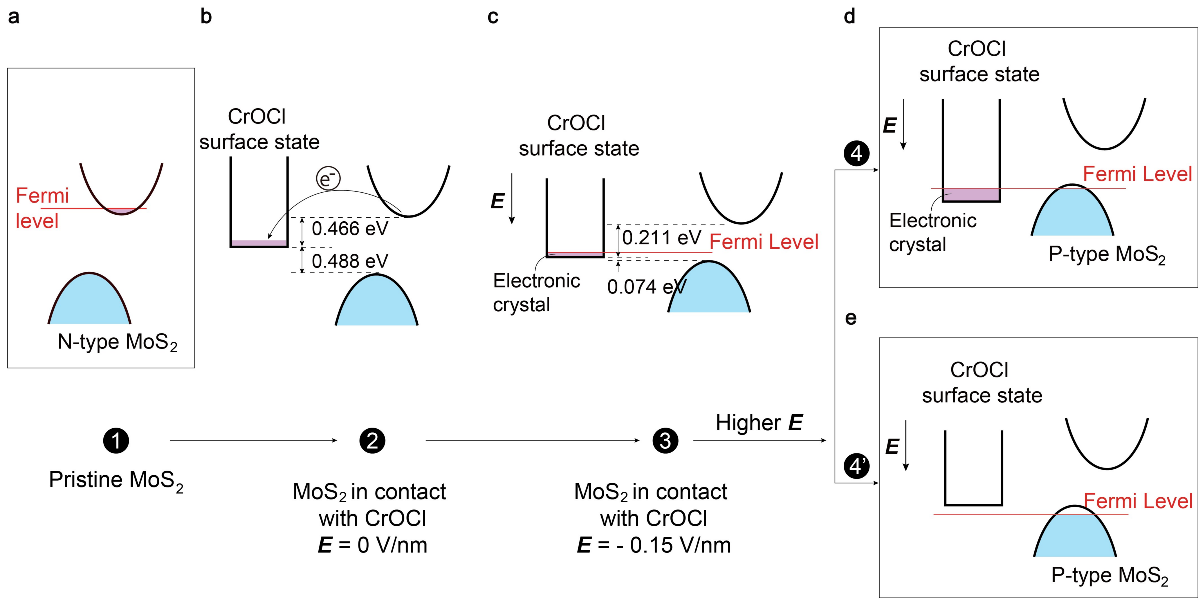

Supplementary Figure 4. Schematic band alignments of MoS_2-CrOCl heterostructures. (a) shows the initial state of a n-type MoS_2. While in (b), in our model, when the 10-layered MoS_2 is interfaced with 5-layered CrOCl without any external electrical applied, the interfacial charge transfer takes place, leading to band re-alignment. Notice that, unlike a trivial charge transfer, here in our system e-e interaction has to be taken into account. And an insulator is formed in the surface state of CrOCl after the charge transfer, since the CrOCl surface state is not conducting. When a finite negative electrical field (negative bottom gate voltage in the experimental configuration) of $\boldsymbol{E}$ = - 0.15 V/nm is applied in (c), the new Fermi level is pulled down to a much lower position as compared to that in (a), which is showing p-type semiconductor characteristics. Notice that, although the whole bands are shifting up in (c), the relative position of the MoS_2 VBM and CrOCl CBM are getting closer. Along with further increasing the amplitude of negative electrical field, the system will end up in two different band alignments, both of p-type as indicated in (d)-(e). (d) shows that the CBM of CrOCl is lower than the Fermi level, while (e) shows a CBM higher than the Fermi level of MoS_2, respectively. The difference between (d) and (e) depend on the detailed self-balance of total charges and nominal doping in each constituent layers, and both scenarios can be possible even though the general behavior of p-type MoS_2 is the same. The number index of 1 to 4 (or 4') are guides for the evolutions of the doping states and band-alignments in (a)-(e).

2. AFM characterization of a typical MoS_2/CrOCl p-FET

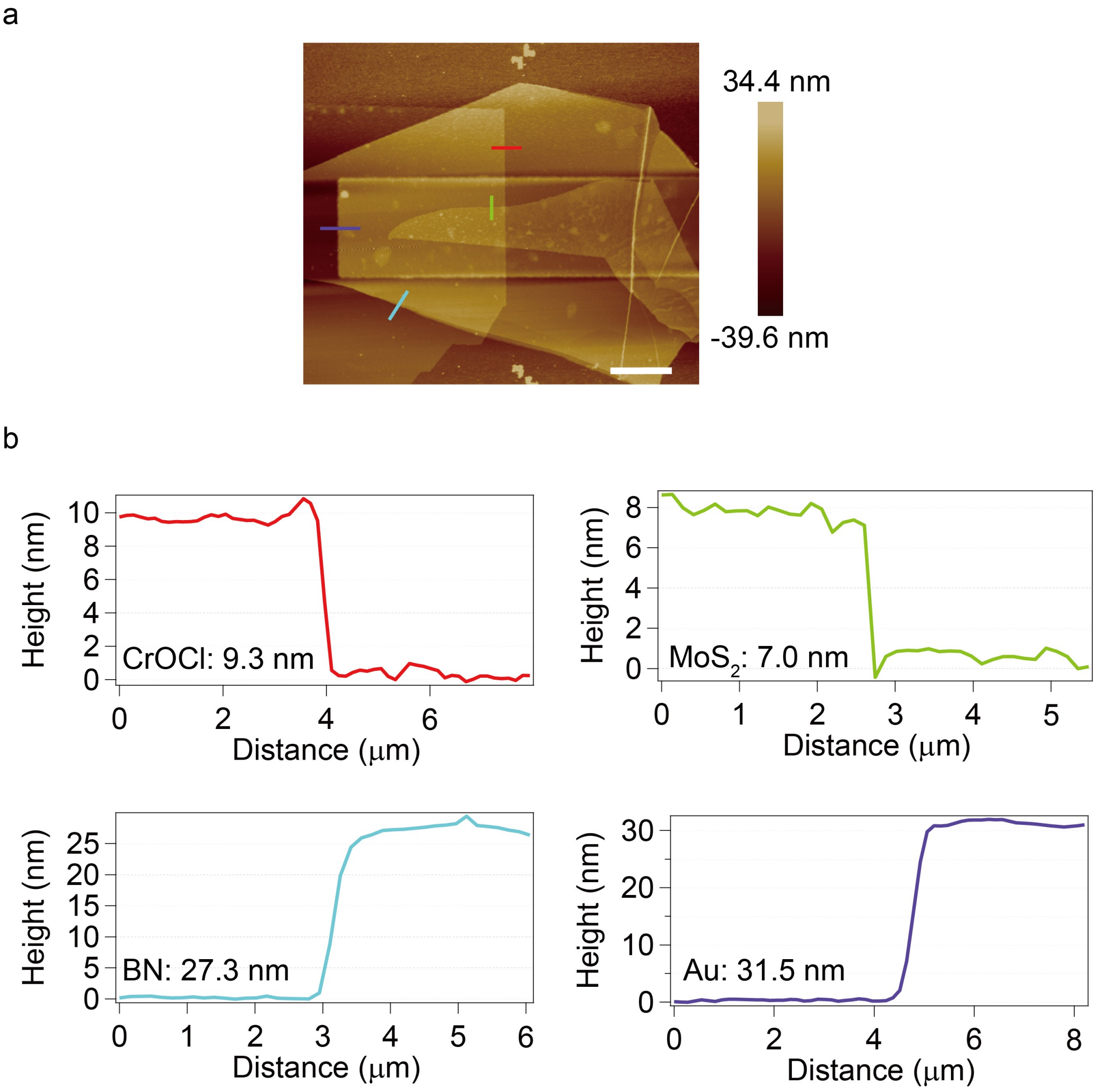

Supplementary Figure 5. AFM characterization of a typical MoS_2/CrOCl p-FET. (a) AFM image of the MoS_2/CrOCl complementary FET shown in Figure 2 in the main text, scale bar is 10 μm. (b) Height profiles of each layer along the solid line in (a).

3. Statistics of transfer characteristics of MoS_2/CrOCl FETs

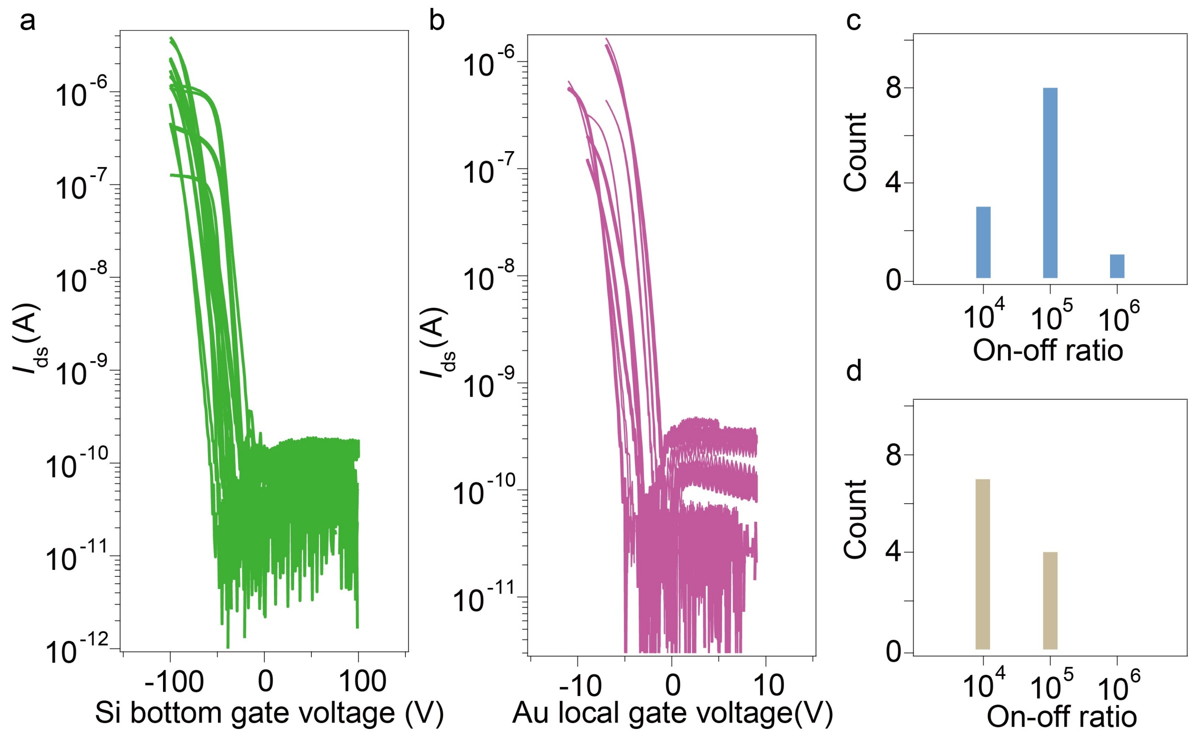

Supplementary Figure 6. Statistics of transfer characteristics of MoS_2/CrOCl FETs. (a) Transfer curves of tested Si gated MoS_2/CrOCl FETs under V_{ds} = 0.1 V, with corresponding statistical histogram of on-off ratio shown in (c). (b) Transfer curves of tested Au gated MoS_2/CrOCl FETs under V_{ds} = 0.1 V, with corresponding statistical histogram of on-off ratio shown in (d).

4. Output characteristics of a typical MoS_2/CrOCl p-FET

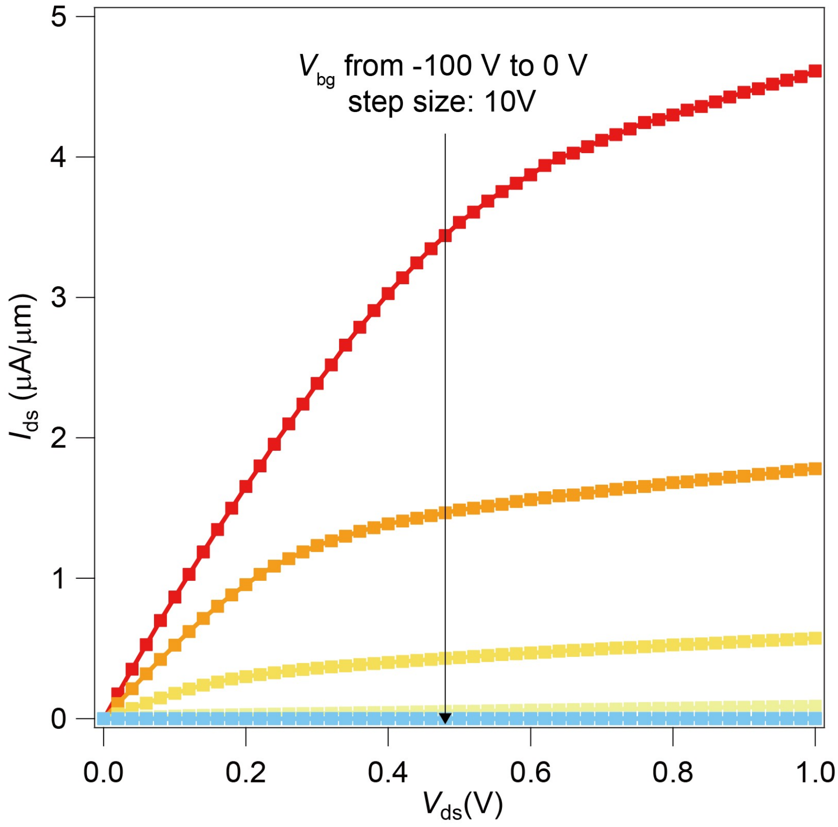

Supplementary Figure 7. Output characteristics of a typical MoS_2/CrOCl p-FET with SiO_2 serving as gate dielectric. Data obtained with 2-probe configuration and standard DC measurements in ambient conditions. Channel size of the tested device is about 6 μm (length) × 1.5 μm (width).

5. KPFM characterizations of MoS_2/CrOCl heterostructures

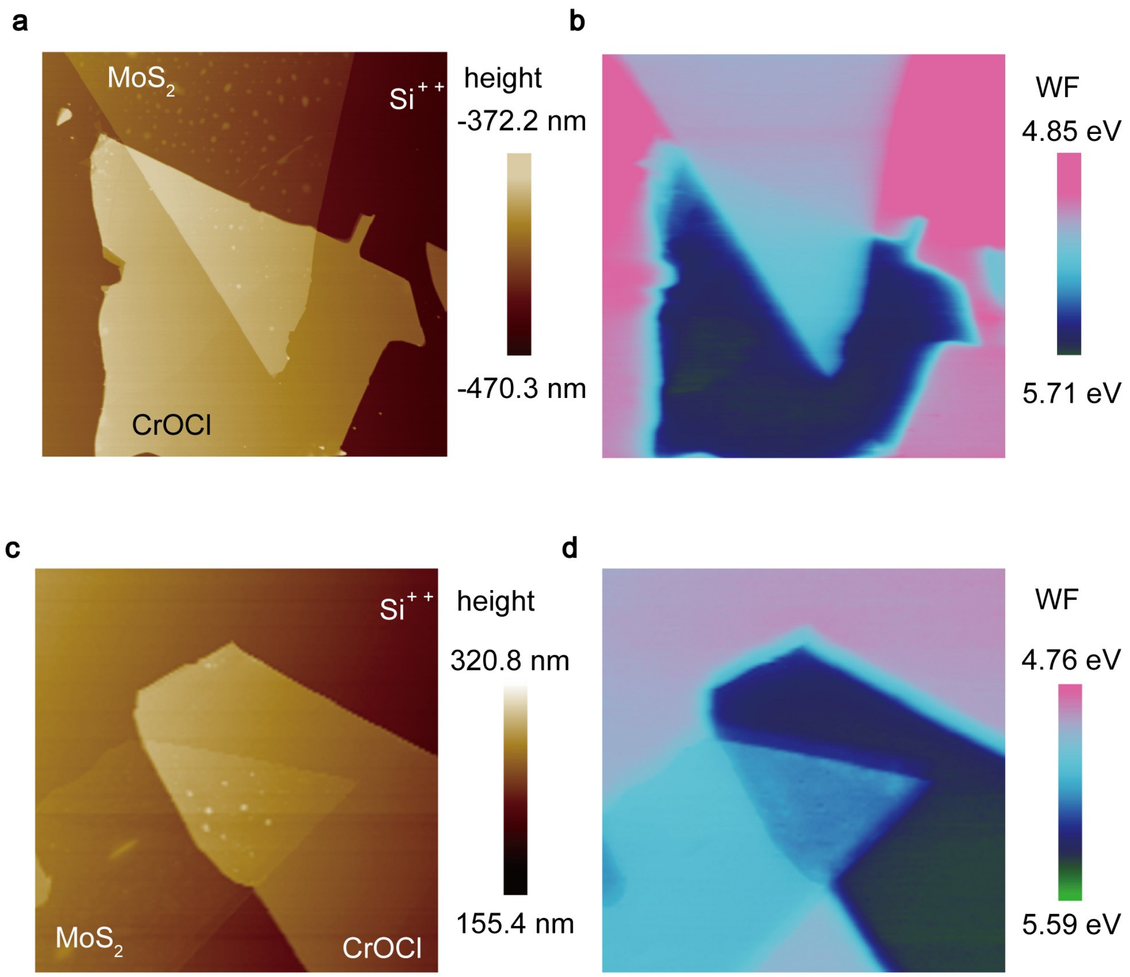

Supplementary Figure 8. KPFM characterizations of two typical MoS_2/CrOCl heterostructures. (a, c) AFM Height image of the two samples. (b, d) Corresponding work function image acquired by KPFM, indicating an increased work function of the MoS_2/CrOCl overlap region, according to the following equation: $-eV_{CPD} = \varphi_{tip} - \varphi_{sample}$,[3] where V_{CPD} is the contact potential difference between the tip and the sample, and φ_{tip} denotes work function of the tip which is extracted by the potential difference with Au (~5.2 eV).

6. Raman spectra of a typical MoS_2/CrOCl sample

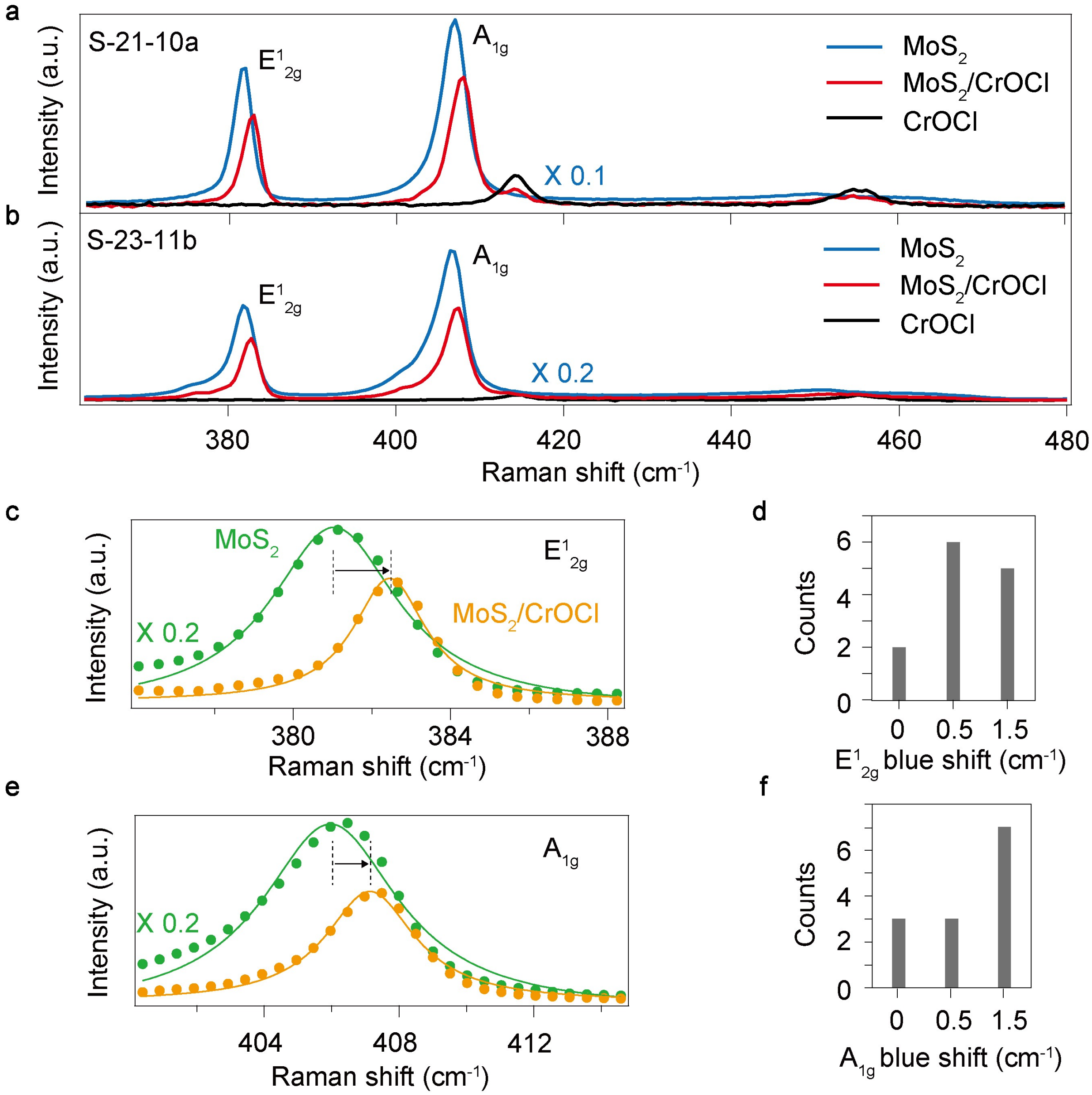

Supplementary Figure 9. Raman spectra of typical MoS_2/CrOCl samples. (a) Raman spectra for three regions of a typical MoS_2/CrOCl sample (No. S-21-10a): few-layer MoS_2, few-layer CrOCl, and few-layered MoS_2/CrOCl overlapping region of the sample. (b) Same data plotted in another sample (No. S-23-11b). As shown in (a), it is seen that the two individual layers exhibit consistent Raman characteristic peaks as compared to the previous reported results[4,5], namely, an in-plane active mode E^1_{2g} at 379 cm^{-1} and an out-of-plane mode A^1_g at 404 cm^{-1} for the few-layer MoS_2, and A_g^2 mode at 411 cm^{-1} and A_g^3 mode at 452 cm^{-1} for the few-layer CrOCl, respectively. Interestingly, compared with few-layer MoS_2, both the frequencies of the E^1_{2g} and the A^1_g modes of MoS_2 in the MoS_2/CrOCl overlapping region

are slightly blue-shifted from 381 to 382.5 cm^{-1} in (c), and from 404 to 405 cm^{-1} in (e), respectively. In (c) and (e), the dotted circles are experimental data, while the solid lines are Lorentzian fit of the measured peaks. Green curves/points correspond to MoS_2 only, while yellow ones correspond to MoS_2 interfaced with CrOCl. It is reported that the bule shift of the $A^1{}_g$ mode can be attributed to a p-doping effect of the few-layered MoS_2.[6,7] We further made a statistic of 13 MoS_2/CrOCl samples whose field effect curves are confirmed to p-type. While most of them showed blue-shifts in both the $E^1{}_{2g}$ and the $A^1{}_g$ modes, and we noticed that, in some rare cases, these heterostructure samples with especially thin CrOCl showed absence of blue-shifts, as indicated in (d) and (f).

7. Electrical performance of $MoSe_2$/CrOCl and WSe_2/CrOCl FET

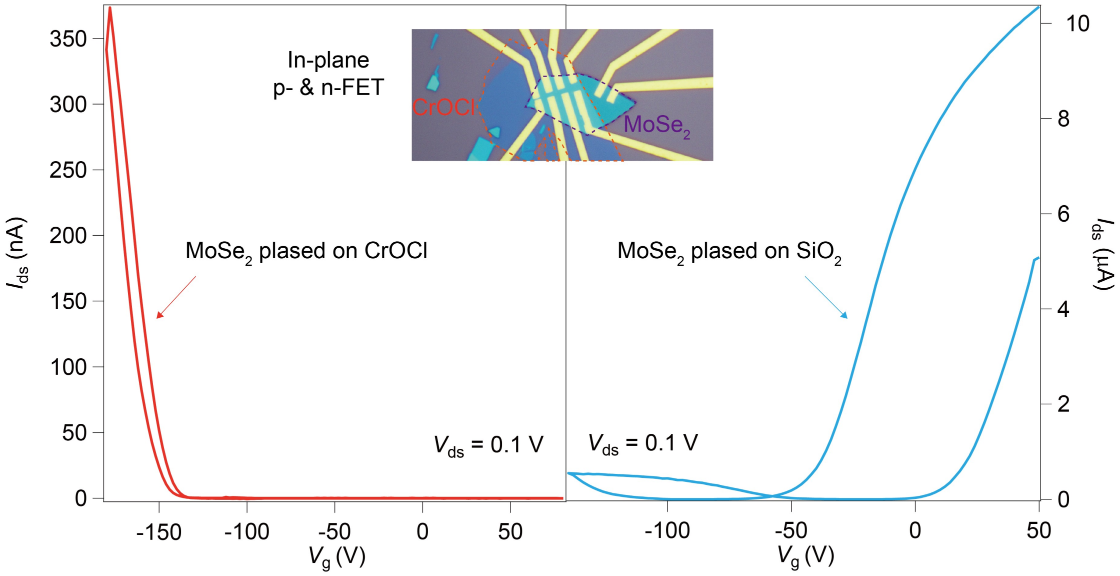

Supplementary Figure 10. Electrical performance of $MoSe_2$/CrOCl FET. Transfer curves measured for a typical $MoSe_2$/CrOCl FET (red line) and a pristine $MoSe_2$ FET (blue line). It is noticed that the hysteresis is much more pronounced when the TMD channel is placed directly onto the SiO_2 substrate, as starkly contrasted to those supported by h-BN as shown in the main text (Fig. 2 for example).

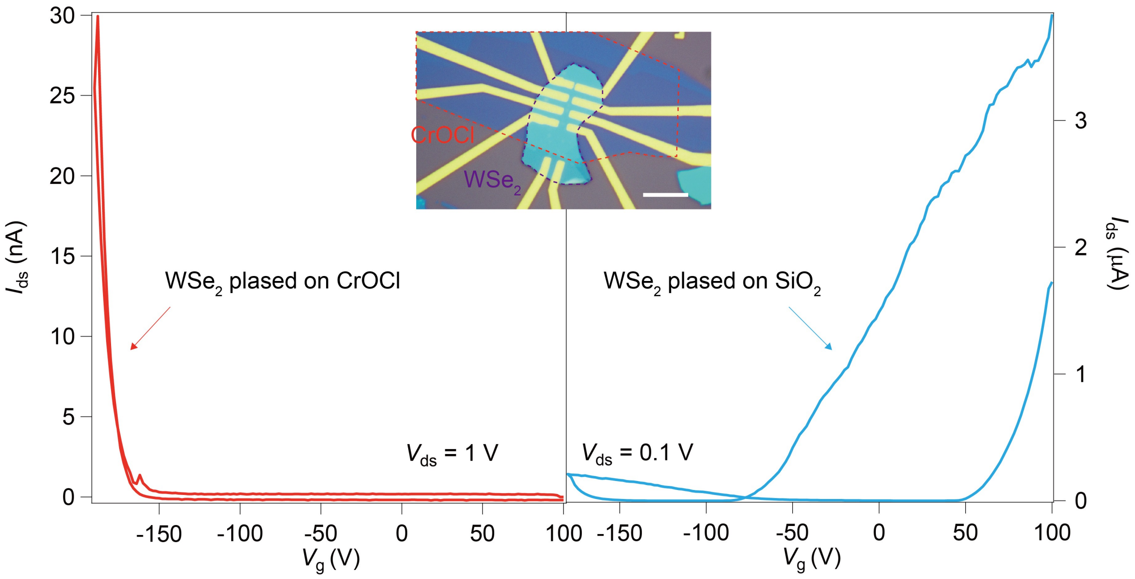

Supplementary Figure 11. Electrical performance of WSe_2/CrOCl FET. Transfer curves measured for a typical WSe_2/CrOCl FET (red line) and a pristine WSe_2 FET (blue line).

8. Electrical performance of MoS_2/CrOCl based planar inverter

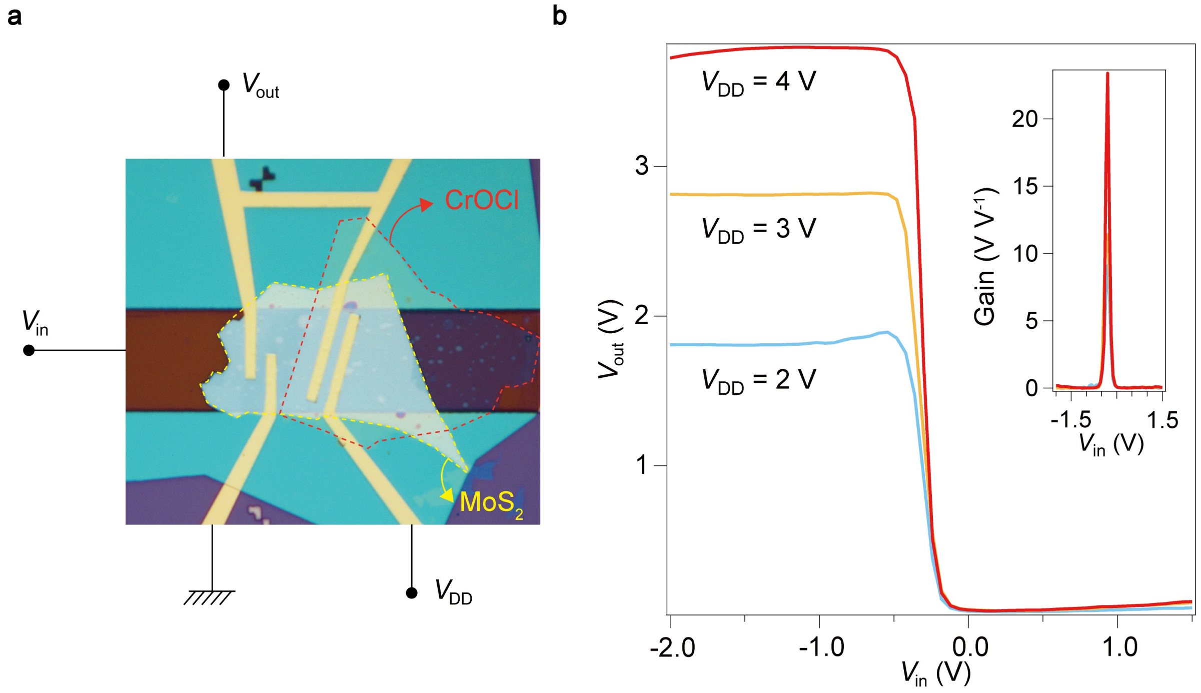

Supplementary Figure 12. Electrical performance of MoS_2/CrOCl based inverter with a conventional planar structure. (a) Optical image of the tested inverter device. (b) V_{in}-V_{out} transfer characteristics of the device with different V_{DD}, inset shows the corresponding voltage gain.

9. Noise margin of the MoS_2/CrOCl based CFET logic inverter

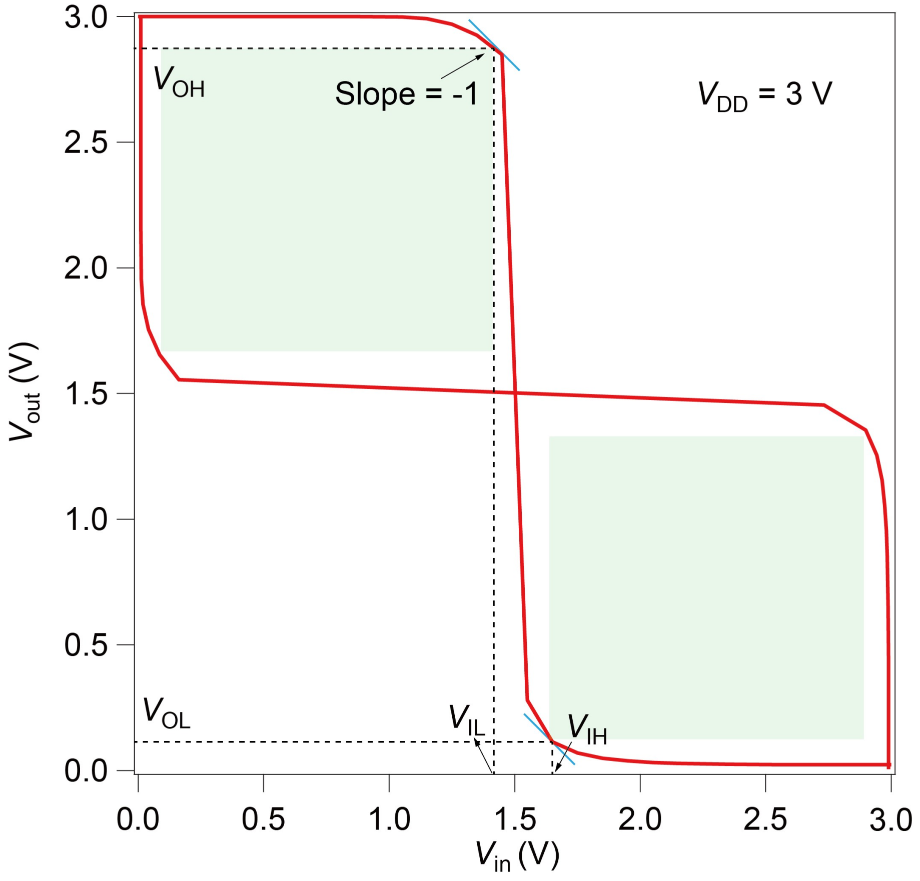

Supplementary Figure 13. Noise margin of the CFET logic inverter under V_{DD} = 3 V. Note that the V_{in} axis of the inverter characteristics was shifted accordingly to make it symmetric with the V_{out} axis in order to extract and compare the noise margins.[8] The V_{OH}, V_{OL}, V_{IL}, and V_{IH} represent the minimum high output voltage, maximum low output voltage, maximum low input voltage, and minimum high input voltage for the inverter, respectively. Noise margins for high input (NM_H) and noise margin for low input (NM_L) are defined as $NM_H = V_{OH} - V_{IH}$ and $NM_L = V_{IL} - V_{OL}$, namely NM_L = 1.22 V and NM_H = 1.29 V, were obtained. And the total noise margin ~83% can be extracted from $(NM_L + NM_H)/V_{DD}$.[9] These values suggest that the CFET logic inverter is highly desirable for securing low sensitivity to noise and disturbances in real circuitries.

10. Dynamic inverting performance of the MoS_2-based CFET inverter

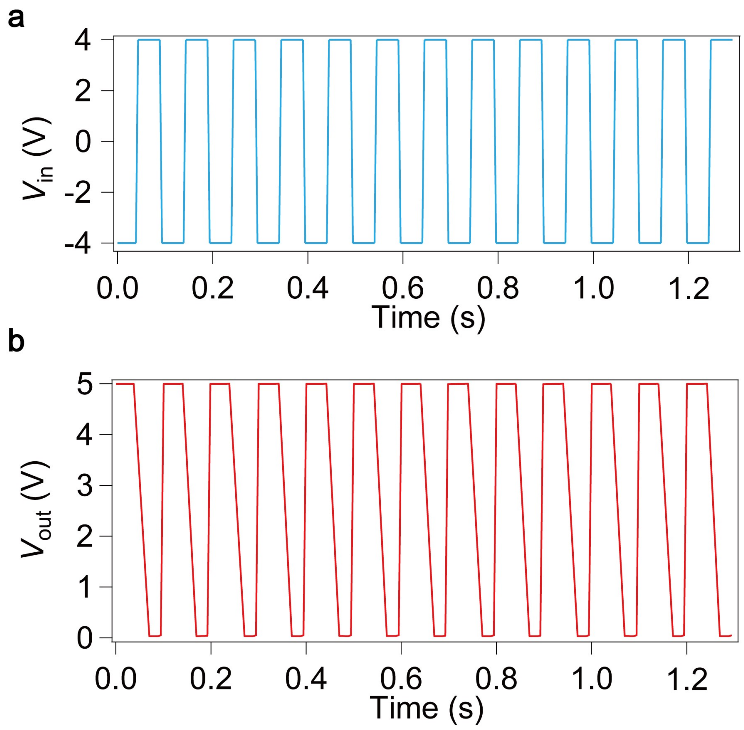

Supplementary Figure 14. Dynamic inverting performance of the MoS_2-based CFET inverter tested at 10 Hz frequency. (a) Input voltage signal. (b) Output voltage signal.

11. Estimation of field effect hole mobilities in typical devices

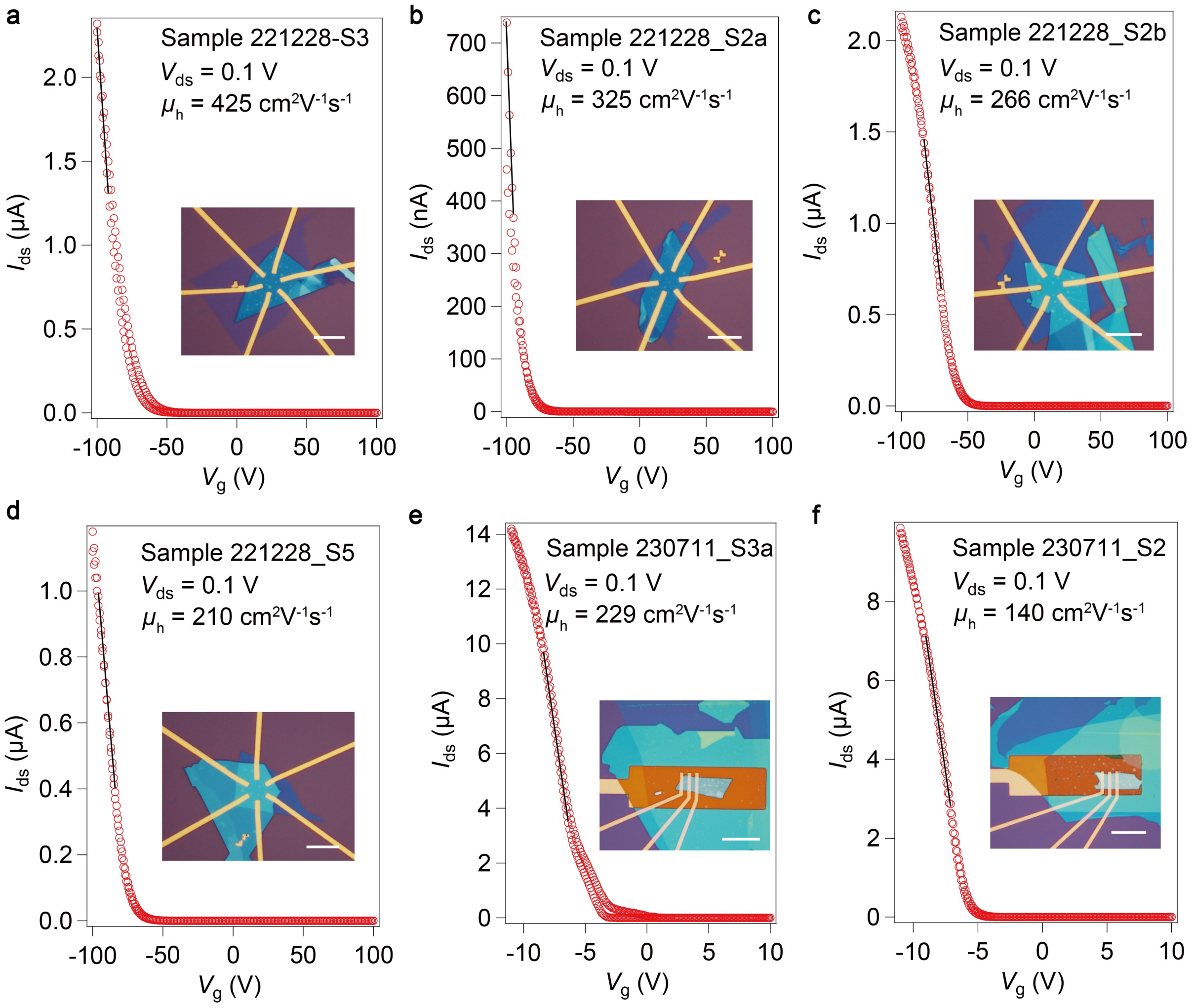

Supplementary Figure 15. Field effect hole mobility extracted from 6 typical MoS_2/CrOCl p-FETs. (a-d) Transfer characteristics of four typical type-I devices with their optical images shown in the insets. (e-f) Field effect curves of two typical type-II devices. These extracted hole mobilities are included in Fig. 2f in the main text.

Supplementary Table 2. Detailed configurations of the devices (images shown in Supplementary Figure 15) tested for the extraction of room temperature hole mobilities.

**Type-I device:** $MoS_2/CrOCl/SiO_2/Si^{++}$ p-FET						
Name of Sample	d_{SiO_2}(nm)	d_{CrOCl} (nm)	L (μm)	W (μm)	$\frac{dI_{sd}}{dV_g}$ (A/V)	μ_h (cm^2 V^{-1} s^{-1})
221228-S3	300	8	6	1.5	1.18×10^{-7}	425
221228_S2a	300	15	6	1.5	8.8×10^{-8}	325
221228_S5	300	30	6	1.5	5.35×10^{-8}	210
221228_S2b	300	30	6	1.5	7.05×10^{-8}	266
**Type-II device:** MoS_2/CrOCl/h-BN/Au p-FET						
Name of Sample	d_{BN} (nm)	d_{CrOCl} (nm)	L (μm)	W (μm)	$\frac{dI_{sd}}{dV_g}$ (A/V)	μ_h (cm^2 V^{-1} s^{-1})
230711_S3a	45	10	2	5	3.48×10^{-6}	229
230711_S2	35	10	2	5	2.57×10^{-6}	140

12. Performances of NAND logic based on MoS_2/CrOCl VIP-FETs

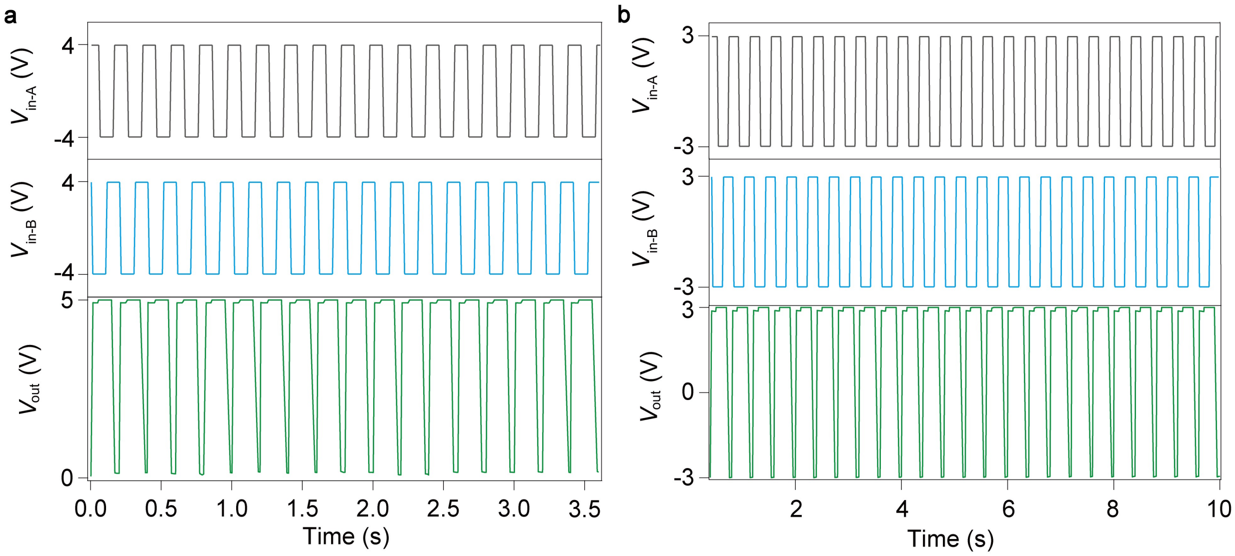

Supplementary Figure 16. Performances of NAND logic based on MoS_2/CrOCl VIP-FETs. (a) and (b) are input and output waveforms of the NAND logic with 14 vdW layers under V_{GND} = 0, and V_{GND}= -3 V, respectively. Depending on the matching of V_{th} between n- and p-FETs, the output waveform may be un-matched (a) or matched (b) with the input waveform. It is preferred to have the operation waves as the one illustrated in (b), in order to power the subsequent stages in circuit designs. It is also noticed that, although we are mainly focused on the CrOCl interfaced TMDs in this work, other layered insulators (or much lower conductivity compared to TMDs at room temperature) such as $Cr_2Ge_2Te_6$ (shown in Supplementary Figure 19), or even TMD/CrOCl heterostructures with different gate dielectrics (Supplementary Figure 20) will give rise to rather different range of V_{th}. It says the fact that one can indeed engineer the V_{th}, to some extent, of the p-type semiconducting channel using the strategy devised in this work.

13. Supplementary Note 2. Discussions of universality of the interfacial coupling induced p-doping effect and the improvement of their electrical performances

In this Supplementary Note, we first discuss the role of layer thickness of MoS_2 in the p-type MoS_2/CrOCl devices. It is found that, when thickness of MoS_2 is lower than a certain value, the p-dope does not take effects. Here in this work, the thinnest device which holds the validity of p-doped behavior is shown in Supplementary Figure 17, with the thickness t of the MoS_2 flake being about 3.2 nm. This might due to the fact that thinner TMDs have larger band gaps which do not fulfill the required Fermi level down-shift as calculated by our DFT results, as illustrated in Supplementary Figure 4. To support this theory, calculated band edge of CBM for CrOCl and VBM for monolayer MoS_2 as a function of electrical fields are shown in Supplementary Figure 18.

Finding other candidate materials other than CrOCl that can also play the role of p-doping TMDs is of importance for future experimental explorations. Indeed, we found that few-layered $Cr_2Ge_2Te_6$ can effectively dope monolayered MoS_2 into a p-type FET, as shown in Supplementary Figure 19. Notably, in this scenario, the on-state threshold voltage V_{th} can also be tuned into different values compared to that observed in TMD/CrOCl devices. This might be an indication that controllable p-doping could be possible by selecting different substrates, which is important in the regard of advanced logic-gate designing.

Interestingly, when we substitute the h-BN dielectric with Al_2O_3 or HfO_2 for these TMD/CrOCl FETs, the general p-type doping behavior do not change, but their V_{th} are effectively changed toward the positive direction of gate voltages (Supplementary Figure 20), yielding the so-called depletion mode, instead of enhancement mode p-FET in most of the cases such as that shown in Fig. 2a the main text. The selection of gate dielectric materials, as well as the selection of different interfacial-coupling layer (CrOCl, $Cr_2Ge_2Te_6$, and etc.) make the future logic applications using these p-FETs devised in this work more applicable.

In addition, we also found that, for the TMD/CrOCl FETs, metal electrodes with typical thickness of Ti/Au ~ 5/50 nm were fabricated by EBL and e-beam evaporator, followed by an annealing under inert atmosphere (320 °C, 5 hours, Ar : H_2 = 30 : 4 sccm) can significantly improve the electrical performances. For example, as shown in Supplementary Figure 21, both MoS_2/CrOCl and $MoSe_2$/CrOCl p-FETs can reach an ON-state current up to ~ 0.2 mA (~ 40 μA/μm, since the width and length of the semiconducting channel is 5 μm, and 3 μm, respectively) at V_{ds} of 2 V.

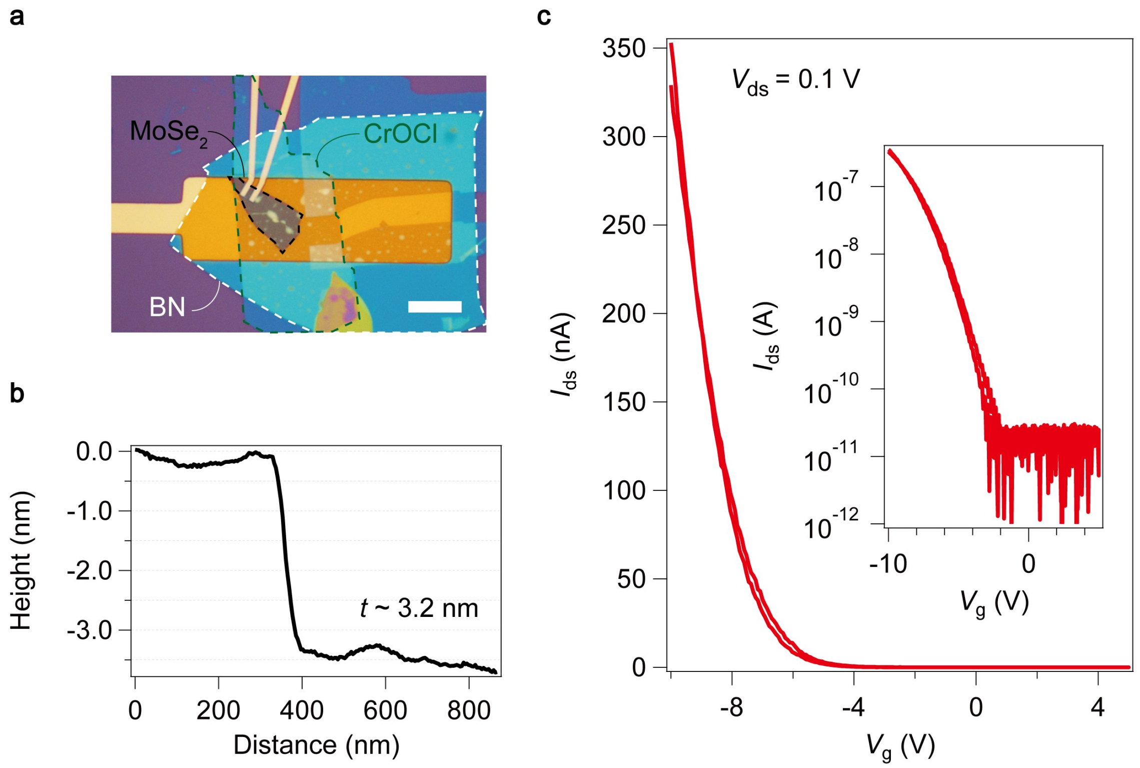

Supplementary Figure 17. Field effect curve of a $MoSe_2$/CrOCl p-FET with t_{MoSe2} ~ 3.2 nm measured at V_{ds} = 0.1 V. (a) Optical photo of the tested device. (b) AFM height profile of the $MoSe_2$ layer in the device. (c) Field effect curve of the device measured at V_{ds} = 0.1 V.

a

b

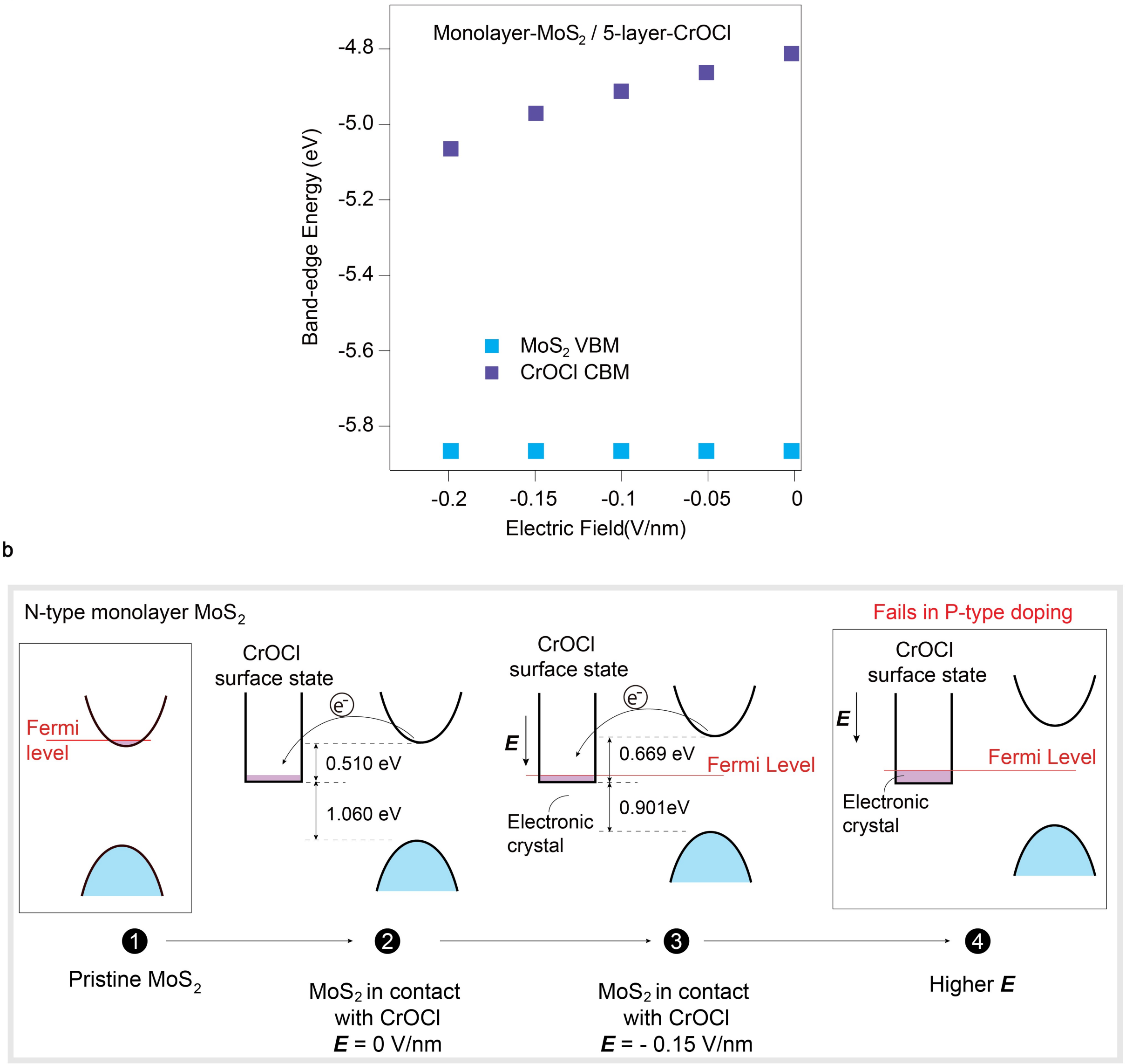

Supplementary Figure 18. Calculated band structure and schematic band alignment of monolayer MoS_2-CrOCl heterostructures. (a) Calculated band edge of CBM for 5-layer CrOCl and VBM for monolayer-MoS_2, as a function of electrical fields. (b) The schematic cartoon shows the failure in p-doping in the case of monolayer MoS_2 in contact with CrOCl, as the Fermi level could not enter the valence band as the multilayer MoS_2 do. This is because of the fact that the monolayer MoS_2 has a much larger band gap and the band edge configuration favourable for the p-doping regime, as shown in Supplementary Figure 4, is not reachable.

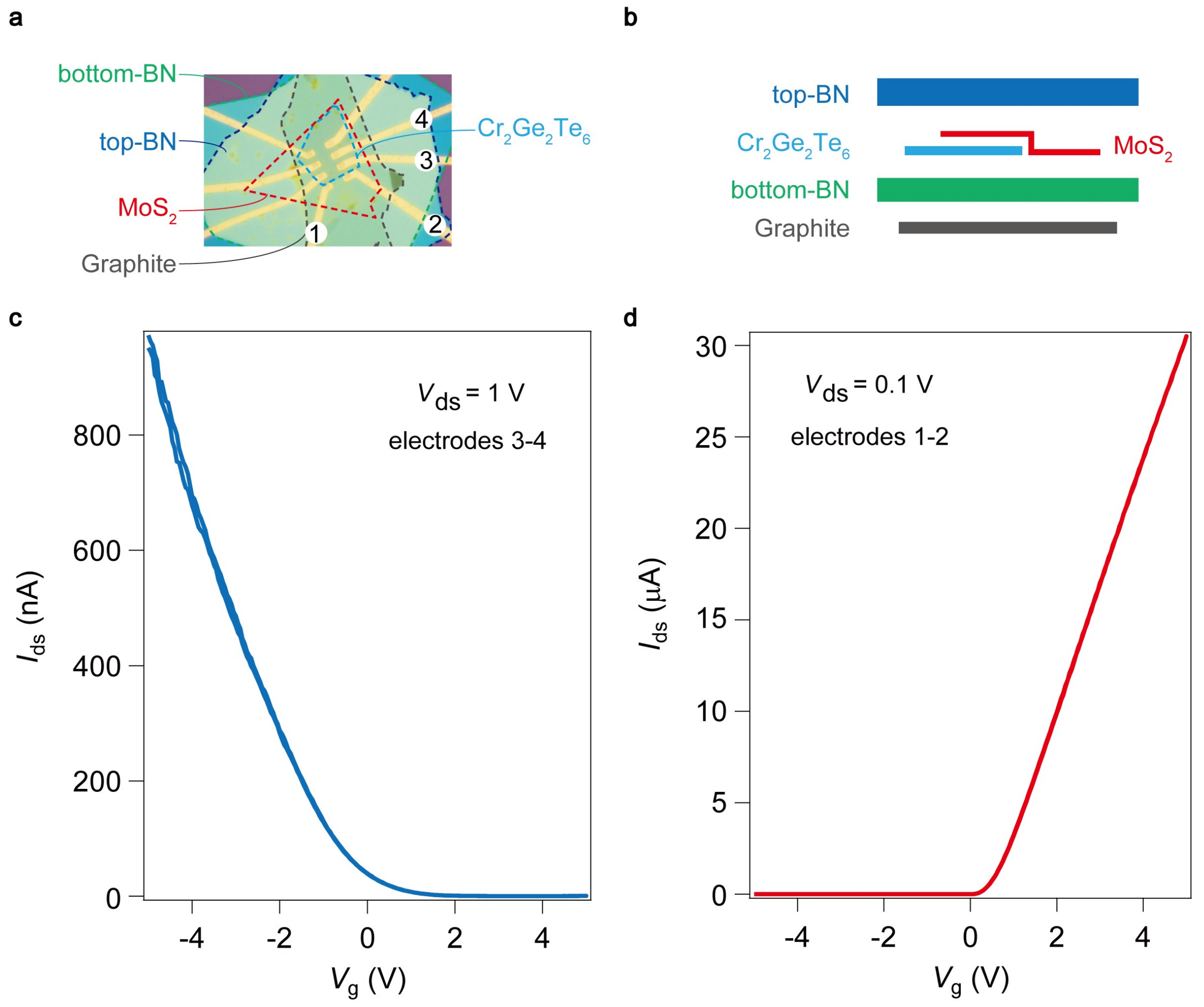

Supplementary Figure 19. Field effect curves of a typical $MoS_2/Cr_2Ge_2Te_6$ p-FET constructed through monolayer MoS_2. (a) Photo image of the $MoS_2/Cr_2Ge_2Te_6$ p-FET. (b) Structure illustration of the device. Field effect property of (c) $MoS_2/Cr_2Ge_2Te_6$ and (d) MoS_2.

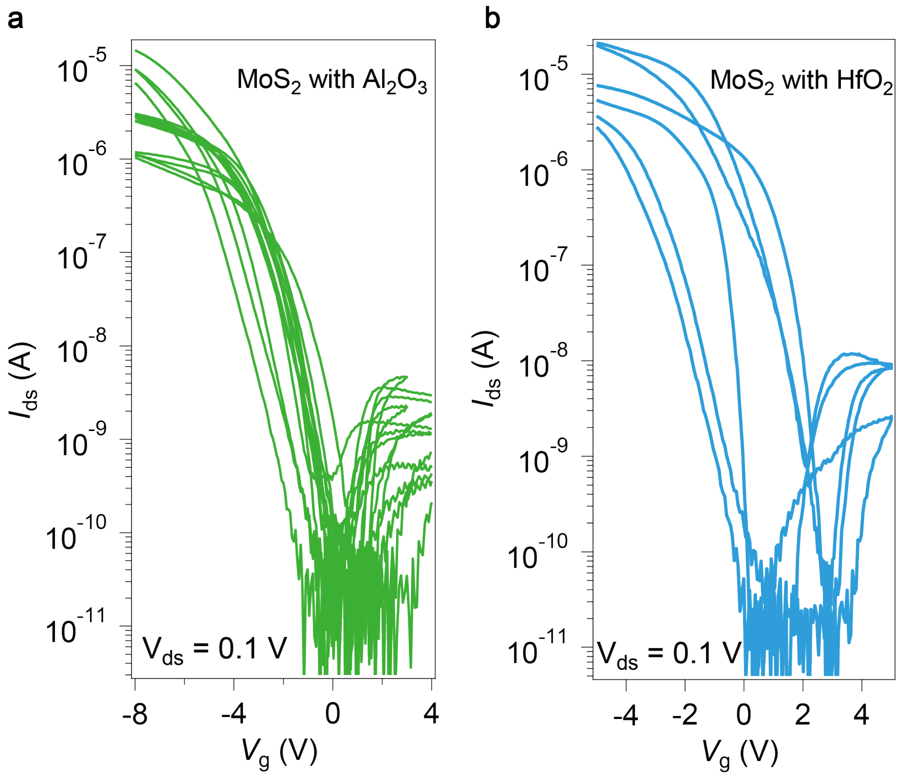

Supplementary Figure 20. Field effect curves of MoS_2/CrOCl p-FETs with (a) Al_2O_3 (20 nm) and (b) HfO_2 (20 nm) served as dielectric layer, respectively.

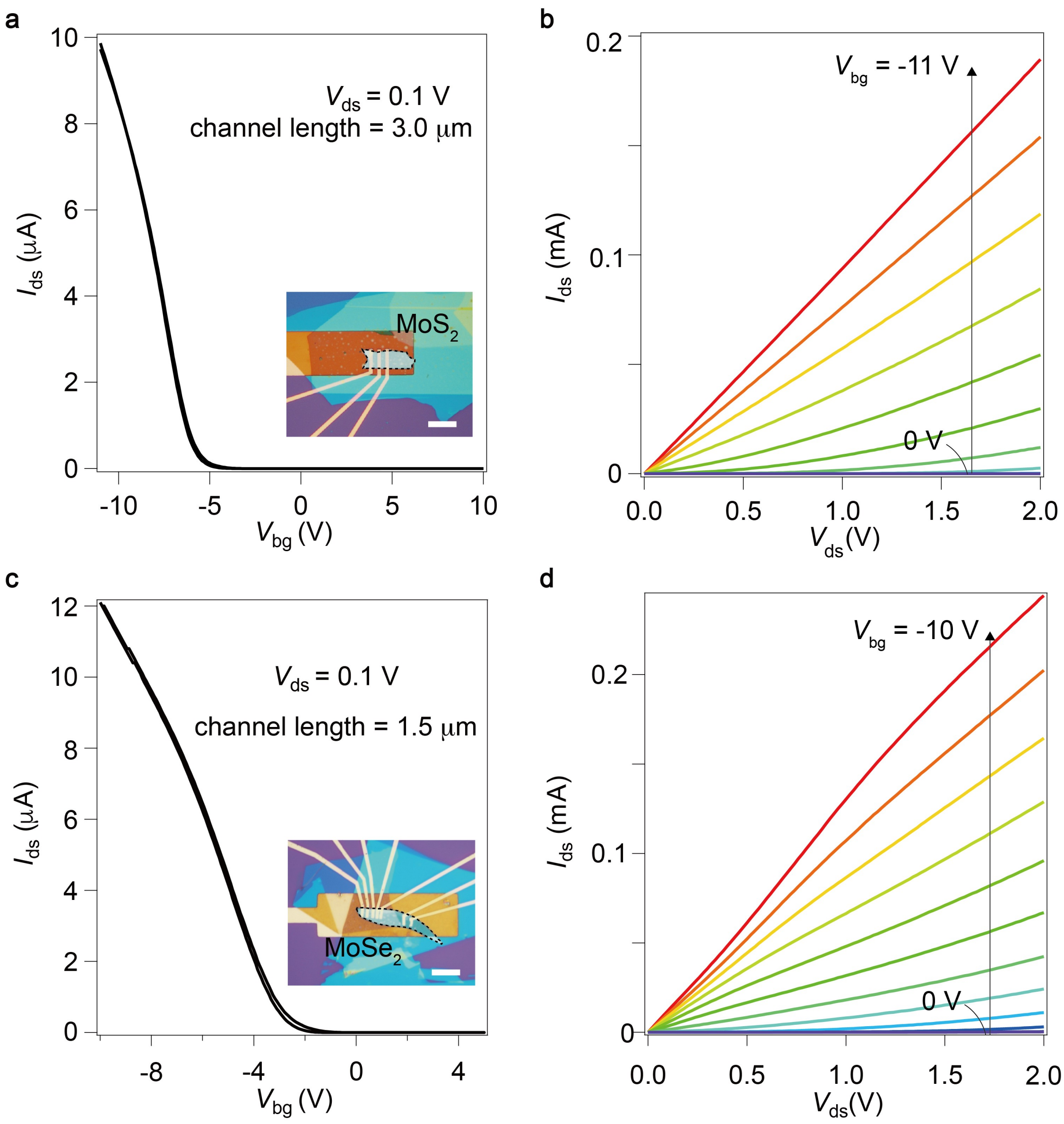

Supplementary Figure 21. Transfer curves and Output characteristics of a typical MoS_2/CrOCl p-FET and a typical $MoSe_2$/CrOCl p-FET. In order to optimize the electrical properties of the TMD/CrOCl FETs, metal electrodes with typical thickness of Ti/Au ~ 5/50 nm were fabricated by e-beam lithography and e-beam evaporator, followed by an annealing under inert atmosphere (320 °C, 5 h, Ar : H_2 = 30 : 4 sccm).

Supplementary Table 3. Improvement of electrical performances by annealing procedure. On state current I_{ON} at V_{ds} = 0.1 V are measured at room temperature, in samples typically of lateral size of 2-3 μm in length and 3-5 μm in width. Annealing process are described in the figure caption of Supplementary Figure 21.

		Cr/Au contacts		Ti/Au contacts	
		Without Anneal	Annealed	Without Anneal	Annealed
MoS_2/CrOCl	Carrier type	P	N/A	P	P
	I_{ON} at V_{ds} = 0.1 V	~ 1 μA	N/A	~ a few nA	~ a few μA
$MoSe_2$/CrOCl	Carrier type	P	N/A	P	P
	I_{ON} at V_{ds} = 0.1 V	~ 300 nA	N/A	~ a few nA	~ 10 μA

14. Supplementary Note 3. Possibility of large-scale production of CrOCl thin films

One of the challenges for future applications based on the VIP-FETs described in this work is the scaling up of these devices. Apparently, large scale production of CrOCl thin layers can be a starting point. In this supplementary note, we discuss the possibility of chemical vapor deposition (CVD) growth of CrOCl thin films.

2D CrOCl nanoflakes were synthesized by an ambient pressure CVD method. The growth was conducted in a 1-m length, 1-inch outer diameter quartz tube heated by a one-zone furnace (MTI, OTF-1200X-III-C). The reaction principle for synthesizing CrOCl involves the hydrolysis of chromium trichloride. The specific reaction equation is as follows: $CrCl_3 + H_2O \rightarrow CrOCl + 2HCl$.

According to this reaction principle, we have devised the following experimental procedure: using chromium trichloride powder as the source of chromium and utilizing air as the source of water. In the initial stages, we employ compressed air as the source of air. Different quantities of $CrCl_3$ powder (Alfa, 99.99%) was used as the solid precursor which was placed under a stacked mica substrate in a quartz boat. Prior to the growth process, the tube of furnace was flushed with high-purity Ar at a rate of 300 sccm for 5 min. Then, 50-200 sccm Ar and 1-10 sccm Air was introduced into the CVD system. The furnace was heated to 600-800 ℃ and growth time was set at 10 min. However, regardless of temperature and gas flow rate adjustments, the resulting outcome remains chromium oxide nanoflakes, shown in Supplementary Figure 22. The results indicate that the water content flow still exceeds our expectations.

In order to reduce the water content to a level conducive for the growth of CrOCl during the experimental process, compressed air was no longer utilized. Instead, the air inlet was slightly opened to allow a minimal amount of atmospheric air to enter the reaction system. Furthermore, to achieve thinner nanoflake thickness, a spatial confinement approach for growth was employed as depicted in Supplementary Figure 23.

The specific experimental procedure was as follows: 50 mg of $CrCl_3$ powder was positioned before a stacked mica substrate in a quartz boat. Prior to the growth process, the tube of furnace was flushed with high-purity Ar at a rate of 300 sccm for 5 mins. Subsequently, 200 sccm of Ar was introduced into the CVD system with an incomplete seal of the quartz tube to permit a small amount of air to enter. The furnace was then heated to 780°C for 78 minutes, with the growth time set at 10 minutes. Once the heating process was concluded, the furnace was allowed to naturally cool down. The CrOCl nanoflakes would be obtained between the two

mica pieces. An optical image of the synthesized CrOCl nanoflakes is depicted in Supplementary Figure 24.

The nanoflakes exhibited a rhombic morphology. To characterize the phase of the nanoflakes, Raman and XRD analyses were conducted. As shown in Supplementary Figure 25 (a), the Raman peaks at 209.5 cm^{-1}, 418.5 cm^{-1}, and 460.5 cm^{-1} corresponds to the A_g mode of CrOCl. The XRD patterns are shown in Supplementary Figure 25 (b). The sample demonstrated a strong preferred orientation, with the intense peaks in the pattern corresponding to the (001) diffraction plane of CrOCl. In summary, CrOCl nanoflakes were successfully synthesized. Different coverage of the growth can be seen in Supplementary Figure 26.

It is thus believed that wafer-scale growth of CrOCl thin films, followed by either further transfer or growth steps of TMDs (and by repeatedly doing so) could lead to larger scale or higher number of stacked layers of VIP-FET arrays, which will be our future studies.

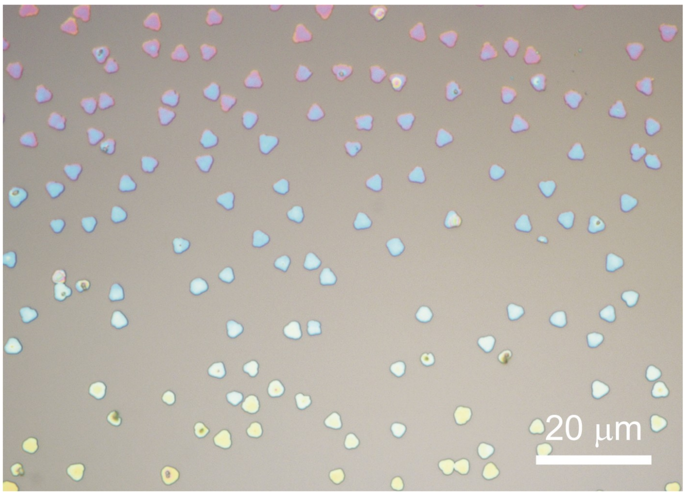

Supplementary Figure 22. Optical images of the obtained chromium oxide (Cr_2O_3).

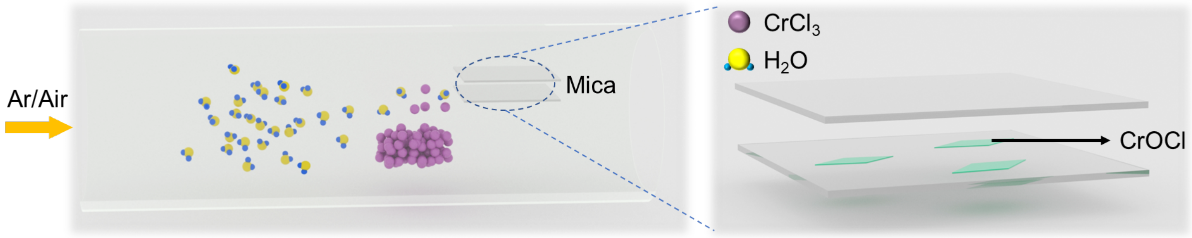

Supplementary Figure 23. Schematic illustration of the CVD setup for growing CrOCl nanoflakes.

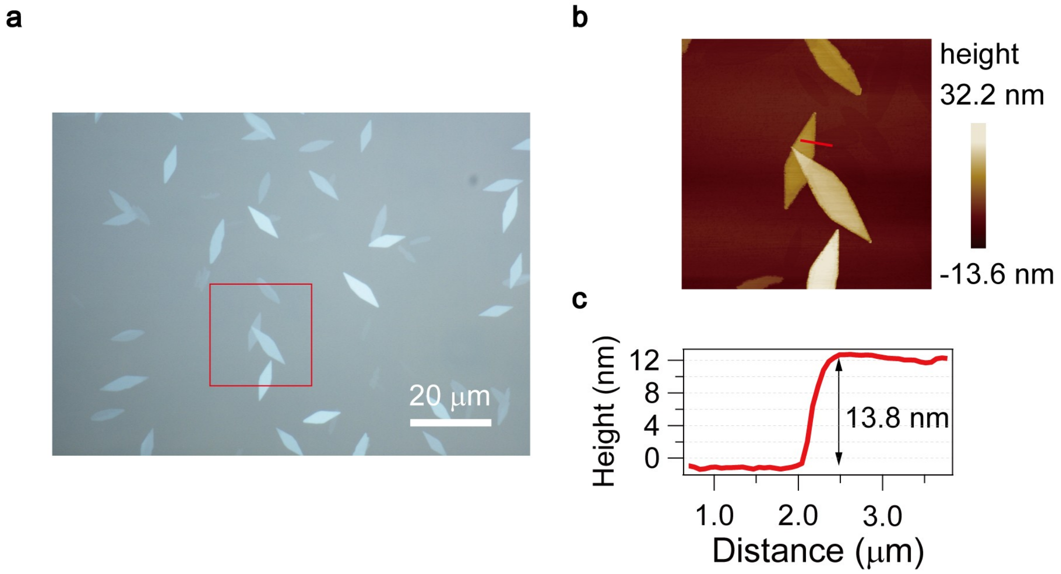

Supplementary Figure 24. Optical images of the obtained CrOCl nanoflakes. (a) Optical micrograph of the obtained CrOCl nanoflakes. (b) AFM height map of the selected area in (a). (c) Height profile along the red solid line in (b).

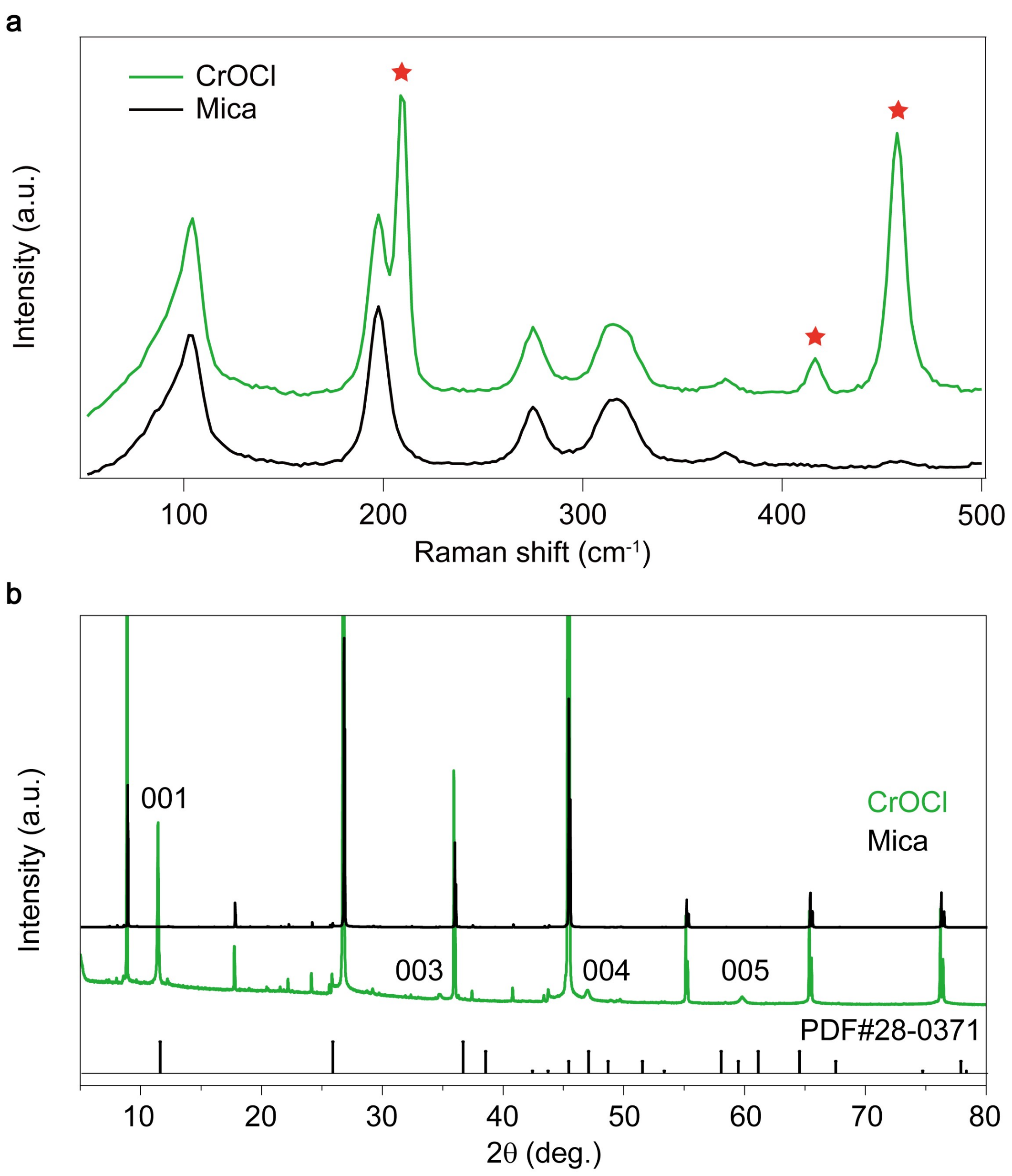

Supplementary Figure 25. Raman (a) and XRD (b) of the obtained CrOCl nanoflakes.

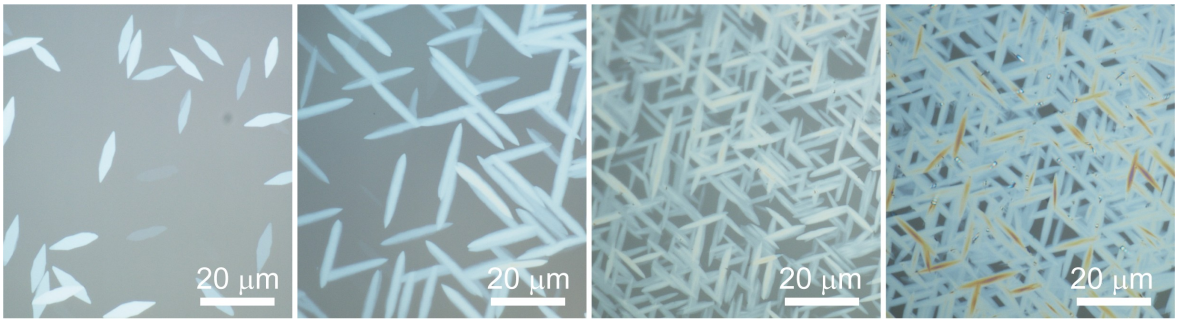

Supplementary Figure 26. Optical images of the obtained CrOCl nanoflakes with different coverage rates. It is believed that wafer-scale growth of CrOCl thin films, followed by either further transfer or growth steps of TMDs (and by repeatedly doing so) could lead to larger scale or higher number of stacked layers of VIP-FET arrays, which will be our future studies.

15. Supplementary Note 4. TCAD Simulations of parasitic capacitances of the VIP-FETs devices

In this Supplementary Note, we investigate the impact of parasitic effects on the electrical performances of the vertically integrated multi-vdW-layer VIP-FETs devices. For simplicity, complementary field effect transistor (CFET inverter) devices with 6 vdW layers (similar to the device shown in Fig. 2c in the main text) were simulated, in order to estimate the influence of parasitic capacitance at different frequencies on the output characteristics of such an inverter using the TCAD tool Sentaurus.

Before the simulation, calibration of the model was necessary. To simplify the simulation, we assumed that both NMOS and PMOS were doped through body defects, with donors in NMOS and acceptors in PMOS. Degradation of mobility due to vertical electric fields were not taken into account, and a constant mobility model was used instead. Physical effects including the quantum potential model, non-local Schottky barrier tunneling in source and drain, and high-field velocity saturation, were considered in our analysis. As shown in Supplementary Figure 27, the simulated transfer curves closely match the experimental results for both PMOS and NMOS FETs, validating the applicability of our models.

Next, we used the parasitic capacitance extraction tool, *i.e.*, the TCAD tool Raphael, to extract parasitic capacitance between metal lines in the inverter. Generally, two scenarios can be possible in device fabrications: 1) metal lines for different channels are designed in a parallel manner; 2) metal lines for different channels are designed in an orthogonally-intersected manner. When metal lines intersect, parasitic capacitance is introduced only at the intersecting nodes between the two metal lines in upper and lower layers. Due to the characteristics of metal line distribution, this capacitance cannot be treated as a simple parallel plate capacitor. Therefore, specialized parasitic capacitance analysis tools like TCAD tool Raphael are needed. In this work, we analyzed the parasitic capacitance introduced in both parallel and intersecting configurations of metal lines, as shown in Supplementary Figure 28. In the case of orthogonally intersected metal lines (Supplementary Figure 28a), four sets of parasitic capacitances are introduced. However, only the capacitance between V_{dd}-V_{out} and V_{ss}-V_{out} affects the inverter's characteristics, while the capacitance between V_{dd}-V_{ss} and V_{out}-V_{out} has no impact on the output characteristics. For the parallelly arranged metal lines (Supplementary Figure 28b), we focused on the configuration where V_{out} and V_{dd} are overlapping through different layers.

According to the results extracted by Raphael, the two sets of capacitance values were almost identical. In the case of intersecting configuration, $C_1 = C_2 = 5.5465 \times 10^{-16}$ farads, while in parallel configuration, $C_1 = C_2 = 1.4 \times 10^{-15}$ farads. It's clear that the parallel configuration introduces larger parasitic capacitance compared to the intersecting ones.

To further analyze the impact of parasitic capacitance on the inverter characteristics, we performed device-circuit co-simulation using the TCAD tool Sentaurus. NMOS, PMOS, and parasitic capacitance were connected in a circuit netlist. We simulated the input-output characteristics of the inverter at different device sizes, metal overlap configurations, and frequencies. It can be seen that for long-channel devices (similar to the parameters in the experimentally tested devices in the main text), the inverter operates normally below a frequency of 1 MHz (Supplementary Figure 29), with or without considering parasitic capacitance, showing identical V_{out}-Time relationships. However, above 1 MHz, the inverter gradually fails, exhibiting noticeable overshooting effects, which are generally caused by the presence of a large gate-source-drain ($C_{g\text{-}s\text{-}d}$) capacitance in the circuit, leading to an output voltage exceeding the logic high level, or falling below the logic low level. At a frequency of 1 GHz (Supplementary Figure 29), the inverter completely fails, and the overshooting-effect dominates. At this point, the presence (absence) of the parasitic effects results in a slow charging (discharging) of the inverter output voltage, and upon further increasing the frequency to, for example, 10 GHz, the impact of parasitic capacitance becomes much more pronounced. Notably, the parallel configuration of metal electrode lines exbibits larger impact of parasitic capacitance, as shown in Supplementary Figure 29.

It is seen that, for the long-channel (a few micron-meters) devices, parasitic capacitance may start to take effects at a frequency of around 1 GHz, affecting the inverter's charging and discharging characteristics. However, due to the large $C_{g\text{-}s\text{-}d}$ capacitance, significant overshooting already occurs around 1 MHz, and the impact of parasitic capacitance is not negligible at this low frequency regime.

To further validate the role of parasitic capacitance, we simulated the characteristics of small-sized (around 100 nm lateral size) devices, as shown in Supplementary Figure 30.

Due to the smaller $C_{g\text{-}s\text{-}d}$ capacitance in small-sized devices, it is observed that the functionality of the inverter prevails up to 10 MHz, an order of magnitude higher than that of the long-channeled devices. At around 100 MHz, impact of parasitic capacitance becomes significant, slowing down the transition rate of the inverter's high and low levels. Furthermore, parallelly arranged inter-layer electrodes manifest larger parasitic capacitance, resulting in a slower level transition rate as compared to the devices with orthogonally intersected inter-layer electrodes. We do observe failure of the inverters with small-size at 10 GHz, as shown in Supplementary Figure 30.

In summary, the impact of parasitic capacitance is expected to occur between the frequency range of 100 MHz and 1 GHz, leading to a slower transition rate between high and low levels of the simulated 6-vdW-layer VIP-FET inverters. Additionally, inter-layer electrodes configured in a parallel manner (Supplementary Figure 29c & 30c) yields larger parasitic capacitances, as compared to the orthogonally intersected ones (Supplementary Figure 29a &

30a). However, due to the large $C_{g\text{-}s\text{-}d}$ capacitance (capacitance from the device itself, not from the parasitic ones from inter-layer arranged electrodes) in large-sized devices, the inverter starts to fail around 1 MHz, making the impact of parasitic capacitance less noticeable. In contrast, for small-sized devices with smaller $C_{g\text{-}s\text{-}d}$, failure occurs around 1 GHz, allowing for a more pronounced observation of the impact of parasitic capacitance. These simulated results will be a guidance for future nano-electronic circuitries made from the reported VIP-FETs technique in this work.

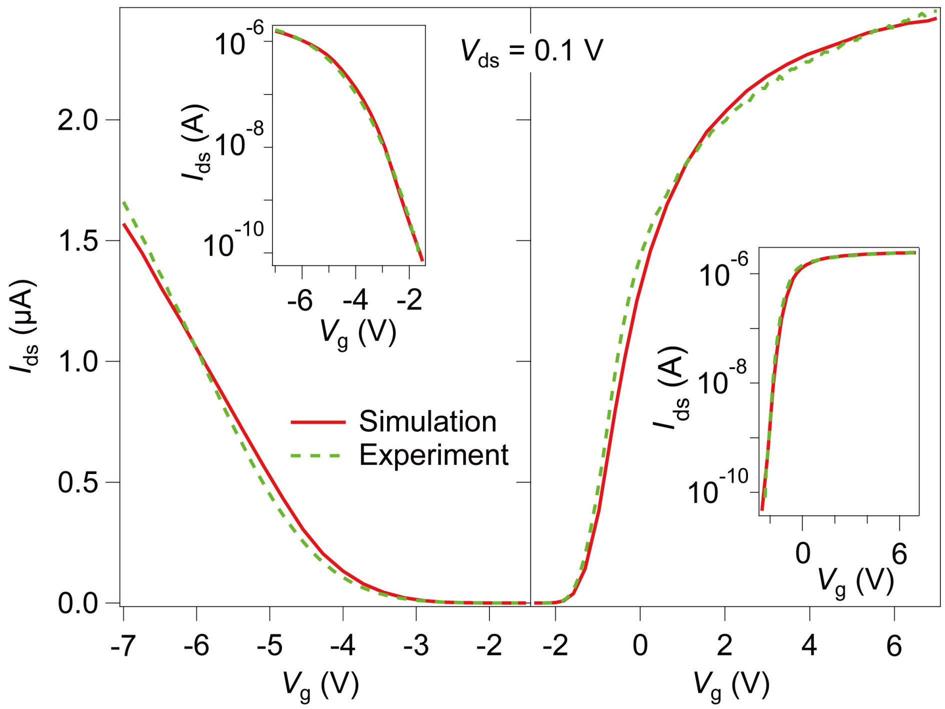

Supplementary Figure 27. Calibration of the model for TCAD simulations. Left: simulated field effect curve (red solid line), according to the experimental data (green dashed line) for the p-FET. Right: simulated field effect curve (red solid line), according to the experimental data (green dashed line) for the n-FET. Insets are the log-scale plot of the corresponding data.

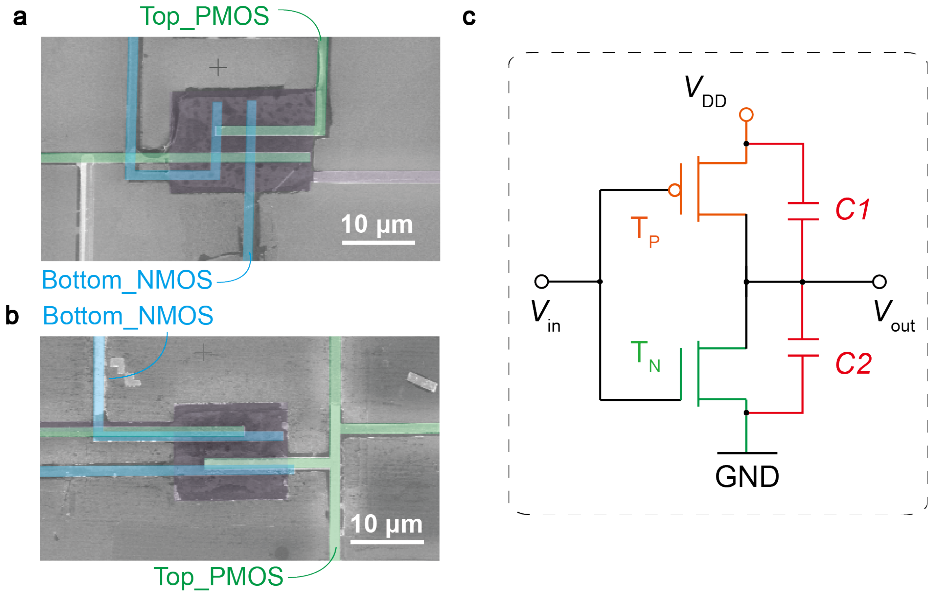

Supplementary Figure 28. Different interlayer arrangements of the electrodes for CFET inverter. The devices are made of 6 vdW layers in a vertical stack, similar to the device described in Fig. 2c in the main text. (a) and (b) are false-colored SEM images for vertical inverter with 6 vdW layers, with their interlayer electrodes constructed in an orthogonally intersected, and a parallel manner, respectively. (c) The schematic drawing of the circuit with two parasitic capacitances labeled as $C1$ and $C2$. T_P and T_N denote p-type and n-type transistors.

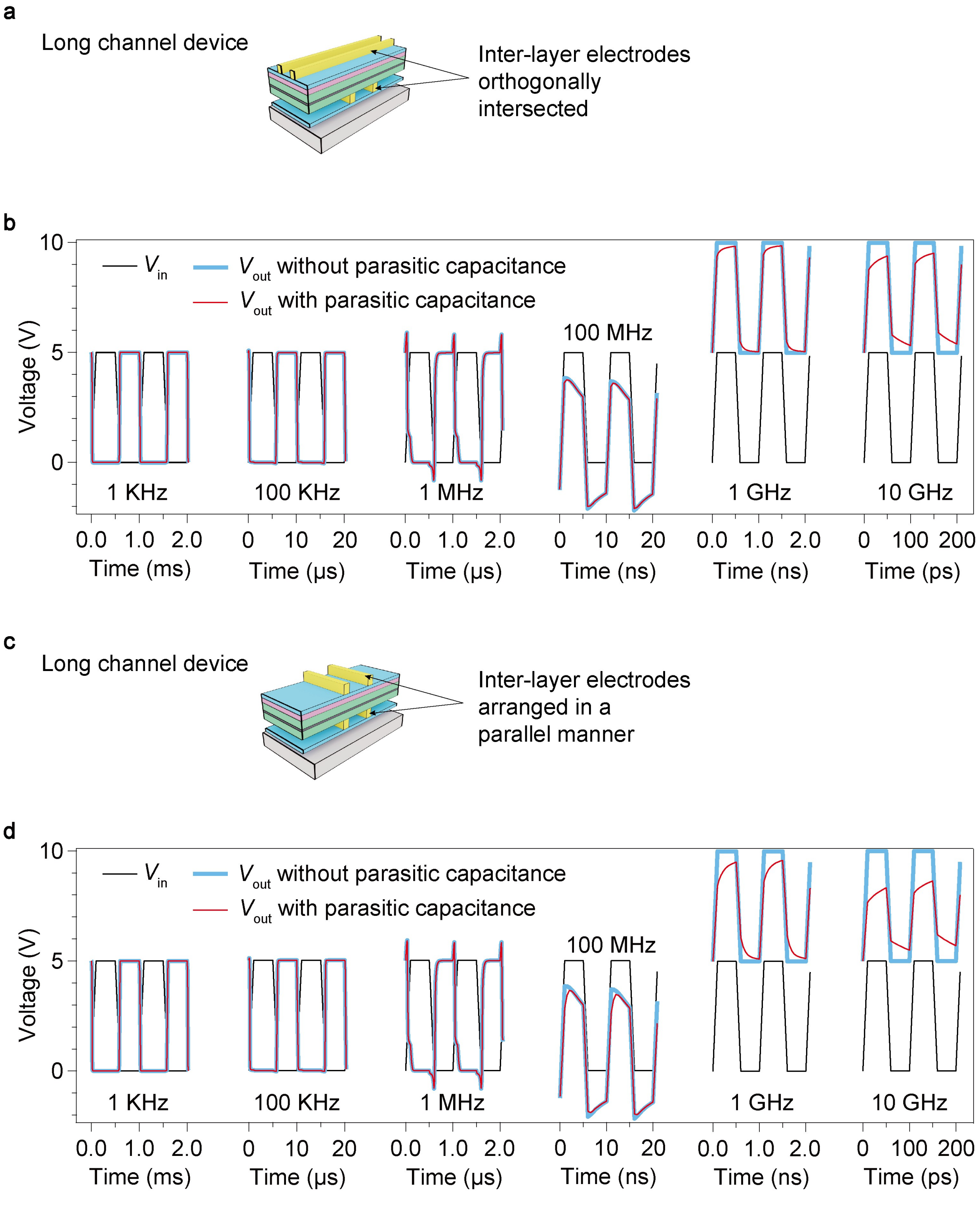

Supplementary Figure 29. TCAD simulations of parasitic capacitances for long channel device. Here 6 vdW layered vertical CFET inverter is considered. The lateral sizes are adopted from experimental device described in Fig. 2c in the main text. (a) and (b) are the cartoon illustration of the vertical CFET inverter with the interlayer electrodes arranged in an intersected manner, and the simulated waveforms at different frequencies from 1 kHz up to 10

GHz with/without the influence of parasitic capacitance, respectively. (c) and (d) are the cartoon illustration of the vertical CFET inverter with the interlayer electrodes parallelly constructed, and the simulated output waveforms at different frequencies with/without the influence of parasitic capacitance, respectively.

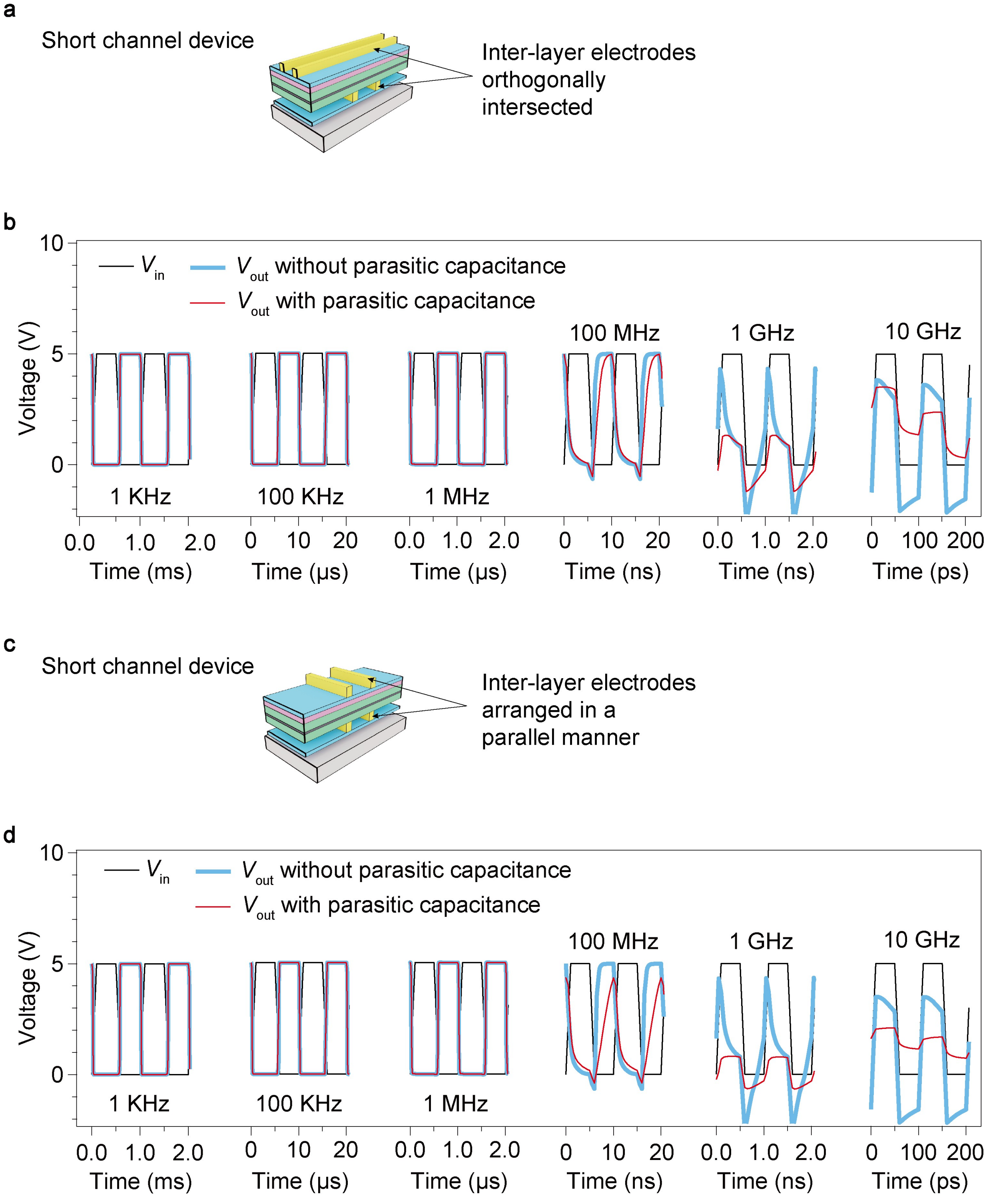

Supplementary Figure 30. TCAD simulations of parasitic capacitances for short channel device. Here 6 vdW layered vertical CFET inverter is considered. Channel length is set to be about 100 nm. (a) and (b) are the cartoon illustration of the vertical CFET inverter with the interlayer electrodes arranged in an intersected manner, and the simulated waveforms at different frequencies from 1 kHz up to 10 GHz with/without the influence of parasitic

capacitance, respectively. (c) and (d) are the cartoon illustration of the vertical CFET inverter with the interlayer electrodes parallelly constructed, and the simulated output waveforms at different frequencies with/without the influence of parasitic capacitance, respectively.

Supplementary References

[1] X. Lu, S. Zhang, Y. Wang, X. Gao, K. Yang, Z. Guo, Y. Gao, Y. Ye, Z. Han, J. Liu, *Nature Communications*, **14***,* 5550 (2023).

[2] M. Li, J. Liu, Manuscript in preparation, (2023).

[3] M.S. Choi, M. Lee, T.D. Ngo, J. Hone, W.J. Yoo, *Advanced Electronic Materials*, **7***,* 2100449 (2021).

[4] H. Li, Q. Zhang, C.C.R. Yap, B.K. Tay, T.H.T. Edwin, A. Olivier, D. Baillargeat, *Advanced Functional Materials*, **22***,* 1385-1390 (2012).

[5] X. Zheng, Y. Wei, Z. Wei, W. Luo, X. Guo, X. Zhang, J. Liu, Y. Chen, G. Peng, W. Cai, S. Qin, H. Huang, C. Deng, X. Zhang, *Nano Research*, **15***,* 9377-9385 (2022).

[6] M. W. Iqbal, K. Shahzad, R. Akbar, G. Hussain, *Microelectronic Engineering*, **219**, 111152 (2020).

[7] Y. Zeng, X. Zeng, S. Wang,, Y. Hu, W. Wang, S. Yin, T. Ren, Y. Zeng, J. Lu, Z. Guo, Y. Xiao, W. Jin, *Nanotechnology*, **31**, 015702 (2020).

[8] L. Tong, J. Wan, K. Xiao, J. Liu, J. Ma, X. Guo, L. Zhou, X. Chen, Y. Xia, S. Dai, Z. Xu, W. Bao, P. Zhou, *Nature Electronics*, **6***,* 37-44 (2023).

[9] L. Kong, X. Zhang, Q. Tao, M. Zhang, W. Dang, Z. Li, L. Feng, L. Liao, X. Duan, Y. Liu, *Nature Communications*, **11***,* 1866 (2020).
